# Essential role of eIF5-mimic protein in animal development is linked to control of ATF4 expression

**DOI:** 10.1093/nar/gku670

**Published:** 2014-08-21

**Authors:** Hiroyuki Hiraishi, Jamie Oatman, Sherry L. Haller, Logan Blunk, Benton McGivern, Jacob Morris, Evangelos Papadopoulos, Wade Gutierrez, Michelle Gordon, Wahaj Bokhari, Yuka Ikeda, David Miles, John Fellers, Masayo Asano, Gerhard Wagner, Loubna Tazi, Stefan Rothenburg, Susan J. Brown, Katsura Asano

**Affiliations:** 1 Molecular Cellular and Developmental Biology Program, Division of Biology, Kansas State University, Manhattan, KS 66506, USA; 2 Arthropod Genomics Center, Kansas State University, Manhattan, KS 66506, USA; 3 Department of Biological Chemistry and Molecular Pharmacology, Harvard Medical School, Boston, MA 02115, USA; 4 USDA-ARS, Hard WinterWheat Genetics Research Unit, Kansas State University, Manhattan, KS 66506

## Abstract

Translational control of transcription factor ATF4 through paired upstream ORFs (uORFs) plays an important role in eukaryotic gene regulation. While it is typically induced by phosphorylation of eIF2α, ATF4 translation can be also induced by expression of a translational inhibitor protein, eIF5-mimic protein 1 (5MP1, also known as BZW2) in mammals. Here we show that the 5MP gene is maintained in eukaryotes under strong purifying selection, but is uniquely missing in two major phyla, nematoda and ascomycota. The common function of 5MP from protozoa, plants, fungi and insects is to control translation by inhibiting eIF2. The affinity of human 5MP1 to eIF2β was measured as being equivalent to the published value of human eIF5 to eIF2β, in agreement with effective competition of 5MP with eIF5 for the main substrate, eIF2. In the red flour beetle, *Tribolium castaneum*, RNA interference studies indicate that 5MP facilitates expression of GADD34, a downstream target of ATF4. Furthermore, both 5MP and ATF4 are essential for larval development. Finally, 5MP and the paired uORFs allowing ATF4 control are conserved in the entire metazoa except nematoda. Based on these findings, we discuss the phylogenetic and functional linkage between ATF4 regulation and 5MP expression in this group of eukaryotes.

## INTRODUCTION

Changes in mRNA translation regulate distinct cellular processes including metabolism, cell migration, cell adhesion, cell growth, cell-cycle control and tumorigenesis (1). An established mechanism of translational control is that of *ATF4* through upstream open reading frame (uORFs). ATF4 is a pro-oncogenic transcription factor that drives transcription of a myriad of genes involved in nutrient uptake, amino acid synthesis, autophagy and inhibition of apoptosis (2). This regulation is manifested by a special arrangement of two uORFs found in the ATF4 mRNA leader region. The ribosome remains linked to the mRNA after translation of the first uORF (uORF1) and gets committed to re-initiate at a downstream ORF. The second uORF (uORF2) normally inhibits downstream re-initiation at *ATF4*. *ATF4* is preferentially translated when the initiating ribosomes bypass the inhibitory uORF (3).

The established trigger of uORF2 bypass and re-initiation at *ATF4* is the inhibition of the activity of eIF2, an initiation factor that recruits the initiator tRNA to the ribosome dependent on bound GTP. eIF2α kinases (eIF2aK), such as GCN2, phosphorylate eIF2 at Ser 51 of its α subunit, thereby inhibiting its activation by guanine nucleotide exchange and inducing *ATF4* translation. Moreover, any perturbation of other eIF activity or expression that results in inhibiting eIF2 can induce translation of *GCN4*, the yeast equivalent of *ATF4*, whose mRNA leader also contains the paired uORFs. For example, overexpression of eIF5, an essential binding partner of eIF2, inhibits the initiator tRNA binding to the ribosome, thereby mimicking the effect of eIF2 phosphorylation and inducing *GCN4* (4). In line with this observation, we recently reported that overexpression of a new translational inhibitor protein, eIF5-mimic protein 1 (5MP1, also known as BZW2), can inhibit eIF2 through a direct competition with eIF5, thereby inducing *ATF4* translation in mouse embryonic fibroblasts with an eIF2α Ser 51-to-Ala mutation (5).

Humans encode two copies of eIF5-mimic proteins (5MP), 5MP1 and 5MP2 (also known as BZW1) (5), which are 70% identical to each other and expressed in cultured mammalian cells at a level stoichiometric to initiation factors (∼50–80% compared to eIF2 levels) (6). Curiously, human 5MP1 and 5MP2 are highly expressed in placenta and bronchial epithelial cells, respectively, where the likelihoods of exposure to human pathogens is also high (BioGPS). Furthermore, 5MP2 is overexpressed in certain types of cancers, and 5MP2 knockdown in salivary mucoepidermoid carcinoma reduces its tumorigenicity, implicating 5MP in tumorigenesis (7). Through the ‘eIF5-mimic’ C-terminal domain (CTD), human 5MP1 interacts with eIF2 as well as eIF3, the multisubunit ribosome-binding factor, just as eIF5 does (5). While 5MP overexpression may induce ATF4 translation through inhibiting eIF2, the role of 5MP in its normal abundance, i.e. through eIF3 or eIF2 phosphorylation, is currently unknown.

Whereas 5MP is clearly not essential for general protein synthesis, most eukaryotes contain a copy of 5MP (8). 5MP homologs are found in all completely sequenced plant, fungal (only basidiomycota or mushrooms) and animal genomes, and are even found in the primitive eukaryote, *Giardia intestinalis* (*lamblia*). However, they are not found in nematodes including *Caenorhabditis elegans*, yeasts (ascomycetes) including *Saccharomyces cerevisiae* or unicellular protists other than *G. intestinalis*. Among insects, 5MP is known as Krasavietz (Kra) in *Drosophila melanogaster* or eIF5C in other species. Kra is expressed at high levels in fly neurons and certain *kra* mutant flies display defective memory and axon guidance (9,10). Kra is also shown to interact with eIF2, as eIF5 does (10). However, the *Kra* null alleles confer severely reduced viability (10,11). Therefore, the role of 5MP in the whole physiology of multicellular organisms remains to be determined. Here, we aim to establish the phylogenetic relationship among 5MP homologs in eukaryotes and to investigate their conserved functions. To study ATF4 regulation by 5MP in metazoa, we also performed RNA interference (RNAi) in the red flour beetle *Tribolium castaneum*.

## MATERIALS AND METHODS

### Phylogenetic and selection analyses

5MP protein sequences were aligned using MUSCLE, a multiple sequence alignment software (12). The phylogenetic analysis based on this data set was performed using a maximum-likelihood approach in PhyML (13). A more detailed description of these analyses is provided in Supplementary text. For selection analysis, we conducted sliding window analyses of d*N*/d*S* (ratio of non-synonymous to synonymous substitutions) along pairwise sequences using JCoDA software (14). d*N*/d*S* ratios >1 are indicative of adaptive (positive) selection, whereas ratios <1 suggest purifying (negative) selection.

### Cloning and expression of 5MP from diverse eukaryotes

To clone 5MP from *T. castaneum, P. triticina, T. aestivum* and *G. intestinalis*, we performed polymerase chain reaction (PCR) with oligos listed in Supplementary Table S1 and appropriately prepared cDNA as template. Several clones were identified and sequenced to check nucleotide replacements and their authenticity derived from polymorphism (Supplementary Tables S2–S5). The details of 5MP cloning and the analysis of clones isolated are described in Supplementary text. The selected clones were transferred to yeast expression vector under the *GAL* promoter. The yeast transformants bearing the resulting plasmids (pEMBL-5MP derivatives) are assayed, as essentially described (5,15) and described in a greater detail under the Supplementary text.

### Protein–protein interaction studies

Preparation of polyhistidine-tagged full-length human 5MP1 and its N-terminal domain (NTD) (aa. 1–245) and CTD (aa. 245–419) constructs is described in Supplementary file. ITC was performed as described previously (16).

### RNAi in *T. castaneum*

RNAi was performed by injecting dsRNA of ∼500–600 bp, corresponding to each half of the 5MP or ATF4 ORFs, into fecund beetles (17). Adult survival, egg laying and hatching, larval development as well as mRNA expression by real time PCR were monitored, as described in the Supplementary text.

## RESULTS

### Phylogenetic and selection analyses of 5MP homologs

**Figure 1.**
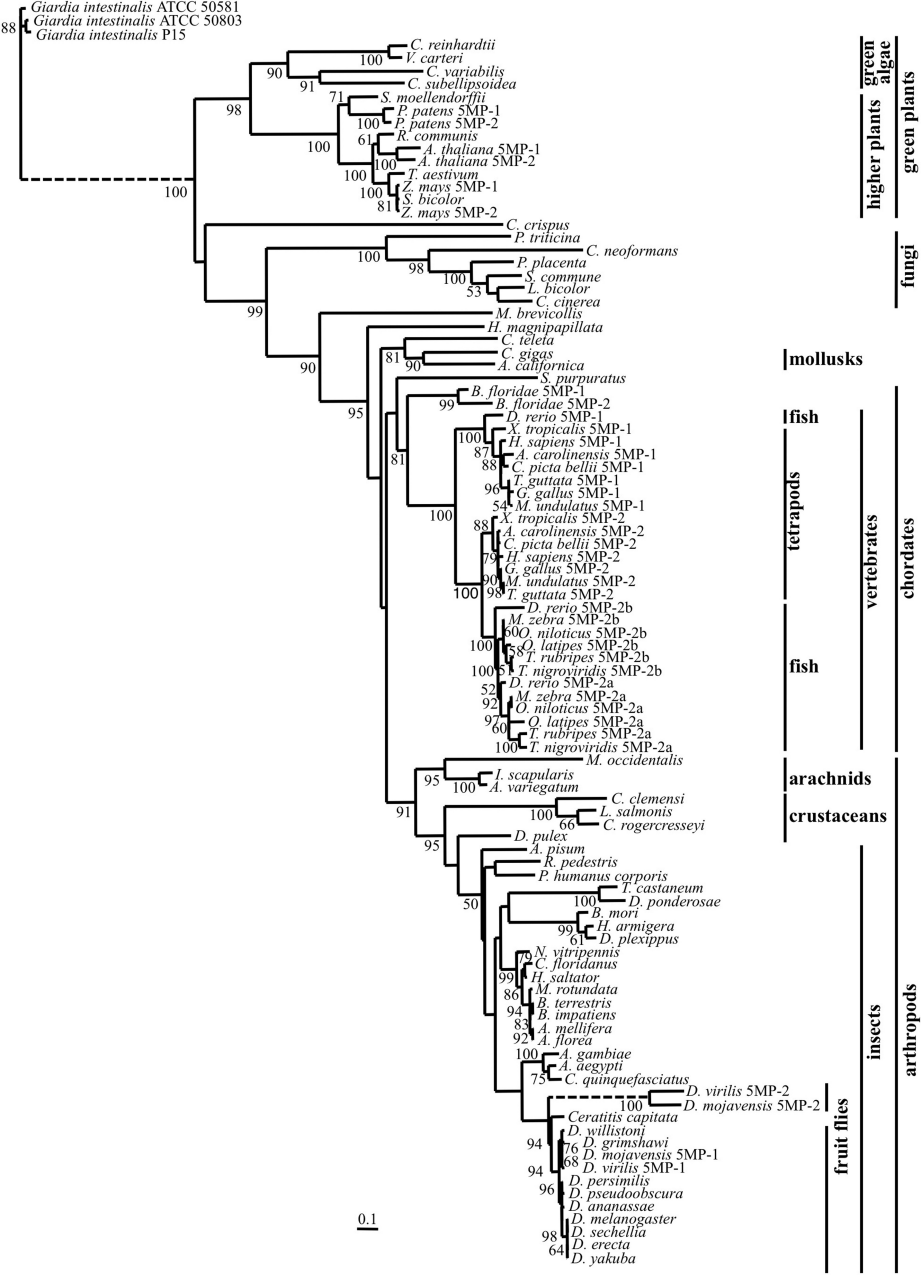
Phylogeny of 5MP homologs. Maximum-likelihood phylogenetic analysis showing the relatedness among 5MP homologs (see Supplementary text for their list). Bootstrap values above 50% are indicated on the branches. This phylogenetic tree was rooted using 5MP from the three *G. intestinalis* strains as outgroup. The dashed lines represent branches that were shortened by a factor of three for presentation purposes.

We used 97 5MP homologs from representative eukaryotic lineages and performed a phylogenetic analysis using a maximum-likelihood approach in order to assess the phylogenetic relationship between 5MP homologs, as well as to analyze the relationship among multiple 5MP copies within a given species. As shown in Figure 1, the 5MP sequences clustered in agreement with the generally accepted phylogeny of the species that were included in this analysis, with green plants and red algae (represented by *C. crispus*) at the base of the tree and the choanoflagellate *M. brevicollis* 5MP forming a sister-branch to the metazoan 5MP clade.

The 5MP tree also showed that 5MP gene duplications occurred independently in plant, chordate and *Drosophila* lineages (see Supplementary text for details). We arbitrarily assigned numbers (1 and 2) and letters (a and b for fish 5MP) for 5MP copies. Two main 5MP clades were formed by vertebrate 5MP proteins: one that contains *D. rerio* and tetrapod 5MP1 and a second clade that contains both vertebrate and fish 5MP2. All fish species surveyed in this study except *D. rerio* contained two 5MP2 copies but lacked 5MP1. However, 5MP1 homologs identified in *D. rerio* and the expressed sequence tag (EST) database of other fish (see Supplementary text) suggest that a duplication of a 5MP gene occurred in an early vertebrate ancestor, and that one copy (5MP1) was subsequently lost in some fish lineages. This second copy was then duplicated in an early ancestor of teleost fish. We also performed sliding window selection analyses with 5MP orthologs and paralogs. In all pairs analyzed, d*N*/d*S* ratios were below 1 (see Supplementary Figure S1 for an example). Thus, the entire 5MP gene can be considered to be under purifying selection.

### 5MPs from different eukaryotes bind and inhibit eIF2 in yeast *S. cerevisiae*

**Figure 2.**
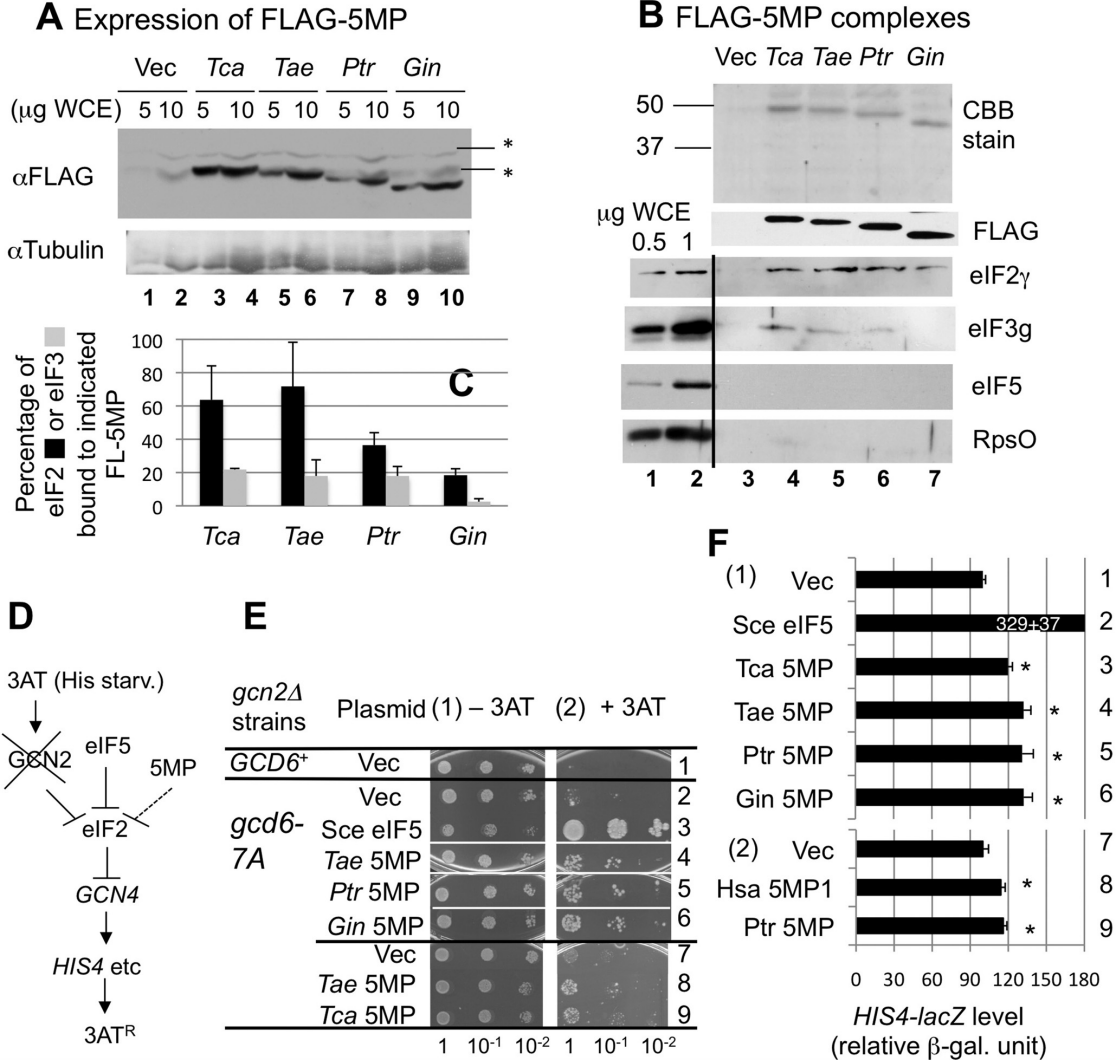
5MPs from diverse eukaryotes bind and inhibit eIF2 in yeast. (A) Expression in yeast *S. cerevisiae*. Indicated amounts of whole cell extracts (WCEs) prepared from KAY33 transformants carrying an empty vector (Vec), or pEMBL-based plasmid coding for *Tca, Tae, Ptr* and *Gin* 5MP and grown in SCGal-Ura medium were subjected for immunoblotting with antibodies listed to the left. Asterisks show cross-reactivity to unknown yeast proteins. (B) Affinity purification. Complexes containing the FLAG-tagged (FL-) 5MPs were purified from KAY33 transformants carrying the plasmids used in (A) as described (15). Note that 5 μl (panel labeled FLAG) or 20 μl (all other panels) of the eluates were analyzed by Coomassie Blue staining and immunoblotting with antibodies raised listed to the right. (C) Graph summarizing the relative amounts of eIF2 or eIF3 associated *in vivo* with FL-5MP, compared to their total amounts in yeast. These values were computed based on molar ratios of eIF2, eIF3 and FL-5MP in the purified fractions, the extent of FL-5MP overexpression compared to FLAG-eIF5 and known stoichiometry of eIF2, eIF3 and eIF5 *in vivo* (18). (D) Model for 3AT resistance by overexpression of eIF5 or 5MP in *gcn2*Δ strain. Dashed stop bar indicates a weak inhibition. (E) Yeast growth assays. Transformants of strain KAY33 (*gcn2*Δ *GCD6*) (row 1) and KAY34 (*gcn2*Δ *gcd6–7A*) (rows 2–9) carrying an empty vector (Vec in rows 1, 2, 7), YEpU-TIF5 (Sce eIF5 in row 3), pEMBL-5MP derivatives expressing 5MP from indicated species were grown in SC-His-Ura medium. Fixed amounts (A_600_ = 0.15) of the culture and its 10-fold serial dilutions were spotted onto the agar plates of SCGal-Ura medium lacking histidine without (panel 1) or with 60 mM 3AT (panel 2) and incubated at 30ºC for 3 and 8 days, respectively. (F) Expression from chromosomally integrated *HIS4-lacZ* in KAY34 (*gcd6–7A*) transformants used in panel E or one carrying pEMBL-FL-5MP1 (5) (Hsa 5MP1) and grown in SCGal-Ura medium for 6 h was presented by relative β-galactosidase units compared to vector control (as 100%). The β-galactosidase units expressed from *HIS4-lacZ* in vector controls are 114.8 and 73.8, respectively, for panels 1 and 2. Asterisk, the *P-*value for the difference compared to vector control is <0.05.

To study the function of 5MP from diverse eukaryotes, we cloned 5MP from *T. castaneum* (*Tca*, red flour beetle), *P. triticina* (*Ptr*, wheat rust), *T. aestivum* (*Tae*, wheat) and *G. intestinalis (Gin)*, representing four major eukaryotic branches, insects, fungi (Basidiomycota), plants and protozoa, respectively (see Supplementary text for details, Supplementary Tables S1 for oligos used for cloning and Supplementary Tables S2–S5 for the analysis of the clones isolated). As shown in Figure 2A, galactose-dependent expression of the cloned FLAG-tagged (FL-)5MP allowed a high level of expression (∼25-fold compared to eIF5) in the yeast *S. cerevisiae* (*Sce*), as seen with FLAG-tagged human 5MP1 (5). Previously, FLAG-human 5MP1 co-immnoprecipitated with eIF2 and eIF3, providing evidence that this protein binds eIF2 and eIF3 (5). To rule out that the ribosome that often binds to the resin mediates the interaction, we eluted the immune complex from the affinity resin. As shown in Figure 2B, top and second panels, we were able to immunopurify FLAG-tagged 5MP samples at a reasonable yield from induced extracts, demonstrating an interaction with eIF2 (Figure 2B, third panel, lanes 4–7; Note the absence of the eIF2γ signal in the mock-purified sample in lane 3). A fraction of the eIF3g subunit also co-immunoprecipitated with *Tca, Tae* and *Ptr* 5MP, but not with *Gin* 5MP (Figure 2B, fourth panel). Because neither eIF5 nor Rps0 co-immunoprecipitated with FLAG-5MP (Figure 2B, panels 5 and 6), the interactions observed here were not bridged by eIF5 or the ribosome, both of which are known to bind eIF2 or eIF3 (15).

Based on the quantification of the levels of overexpressed FLAG-5MP relative to FLAG-eIF5 and the known stoichiometry of eIF2 or eIF3 to eIF5 in yeast (18), ∼20–70% of eIF2 and ∼20% of eIF3 are associated with FLAG-5MP from the different species, though *Gin* 5MP did not bind yeast eIF3 (Figure 2C). Core eIF3 subunits, a, b, c, g and i (19) were conserved in all the eukaryotes we examined, except for *G. intestinalis*, which we found to carry b, c, g and i (Supplementary Table S6). The lack of interaction between yeast eIF3 and *Gin* 5MP may reflect the large evolutionary distance of eIF3 between *S. cerevisiae* and *G. intestinalis* (Supplementary Table S6).

Yeast can overcome growth inhibition by 3-aminotrizole (3AT), an inhibitor of His3p enzyme, by activating translation of the mRNA encoding the transcription factor Gcn4p, a master regulator of general (amino acid) control response responsible for induction of 539 genes induced by 3AT in *S. cerevisiae* (20). Similar to *ATF4*, this translational regulation requires uORFs present in the *GCN4* mRNA leader (see Introduction). Gcn2p-catalyzed phosphorylation of eIF2 or eIF5 overexpression inhibits the binding of initiator tRNA to the ribosome, thereby inducing *GCN4* translation in the absence of starvation signals (Figure 2D). Human 5MP1 overexpression from the *GAL* or *SUI1* promoter can induce *GCN4* translation under non-inducing conditions, when a *gcd6* mutation altering eIF2Bϵ reduces eIF2:GTP:Met-tRNAi ternary complex (TC) levels (5). Similarly, galactose-dependent expression of 5MP homologs allowed *gcn2*Δ*gcd6* cells to grow better in the presence of 3AT (Figure 2E), with an attendant increase in *HIS4(-lacZ)* expression governed by Gcn4p (Figure 2F, panel 1). Because 3AT resistance and *HIS4* expression are the hallmark of Gcn4p-dependent general control response (21), these results suggest that 5MP can inhibit eIF2, thereby inducing *GCN4*. Importantly, the level of *HIS4* induced by the tested 5MP species is equivalent to that caused by human 5MP1 (Figure 2F, panel 2). Thus, we conclude that the 5MP interaction with eIF2, as observed in Figure 2B and C, does have a physiological effect, albeit a weak one (dashed line in Figure 2D).

### 5MP1 interacts with eIF2β at a similar affinity to eIF5 interaction with eIF2β in humans

**Figure 3.**
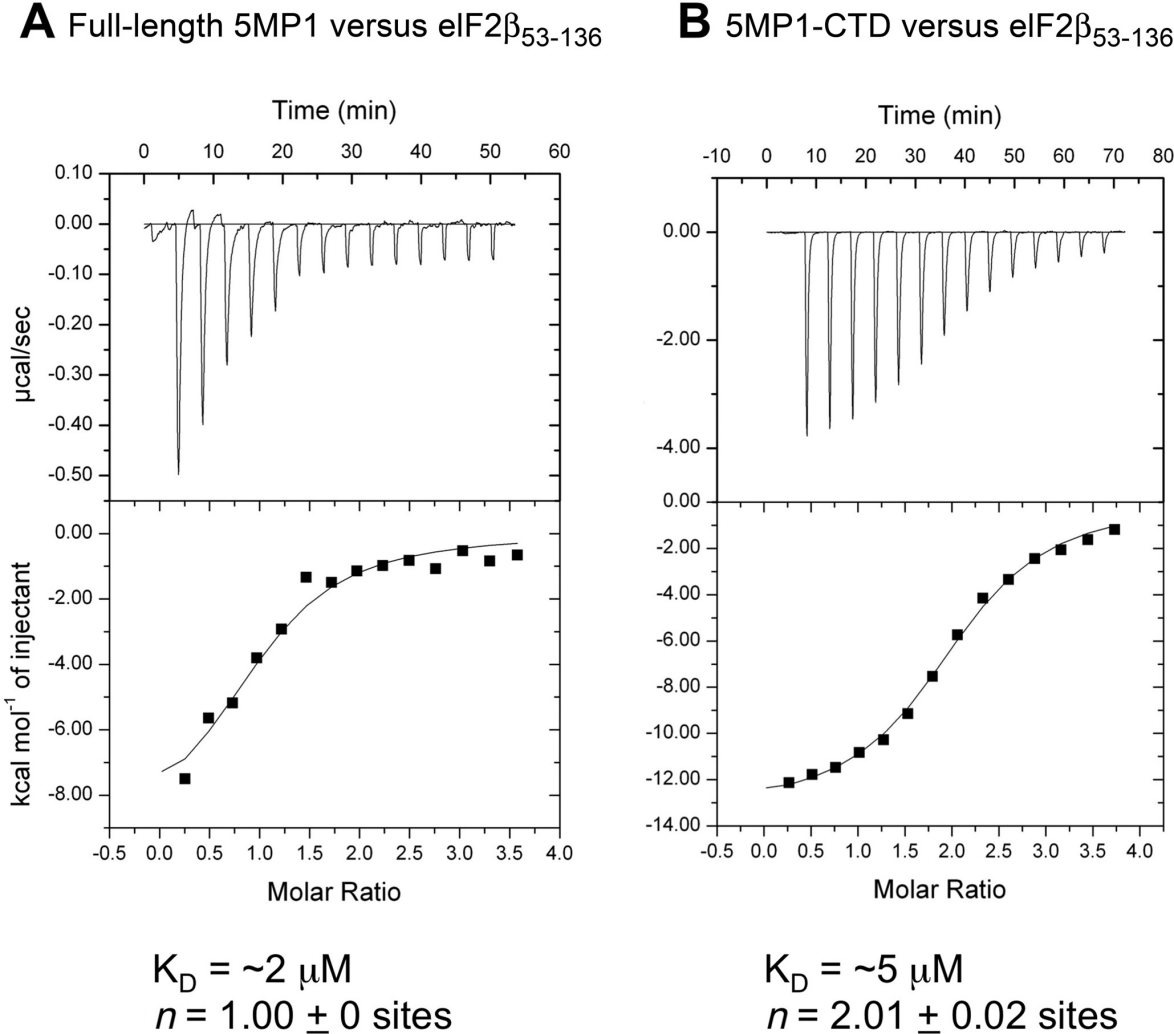
ITC analysis. A solution of eIF2β_53–135_ was injected with full-length human 5MP1 (A) or 5MP1-CTD (B).

Having observed a weak physiological effect of 5MP from various species including a fungus (Figure 2), we considered the possibility that the interaction between eIF2 and 5MP is equivalent to that between eIF2 and eIF5, when measured using proteins from the same species. To test this, we measured the affinity between human 5MP1 and human eIF2β carrying the major competitive binding site for 5MP1 (5). Our previous isothermal titration calorimetry (ITC) analysis showed that the K_D_ between the human eIF5-CTD and human eIF2β_53–136_ carrying two K-boxes, which are the major determinants of interaction with eIF5 (22), is ∼4 μM (16). As shown in Figure 3A, the K_D_ for full-length human 5MP1 and eIF2β_53–136_ was measured here at ∼2 μM. Likewise, the K_D_ for human 5MP1-CTD (similar to eIF5-CTD) and eIF2β_53–136_ was measured at ∼5 μM (Figure 3B). In contrast, we were not able to obtain a binding affinity between 5MP1-NTD and eIF2β_53–136_, in agreement with the idea that the aromatic and acidic amino acid (AA-) boxes present in the 5MP1-CTD that are shared with the eIF5-CTD is the major eIF2β-binding site (5). Interestingly, the stoichiometry for full-length 5MP1 and 5MP1-CTD binding to eIF2β_53–136_ was measured at 1:1 and 1:2 in this assay, respectively (Figure 3). Thus, the role for 5MP-NTD was suggested for the first time as promoting stoichiometric binding to its substrate, eIF2β. These data together reinforce that 5MP is able to compete effectively with eIF5 for the substrate, eIF2 (5).

### Paired uORFs potentially allowing ATF4 regulation are conserved in all metazoa except nematoda

**Figure 4.**
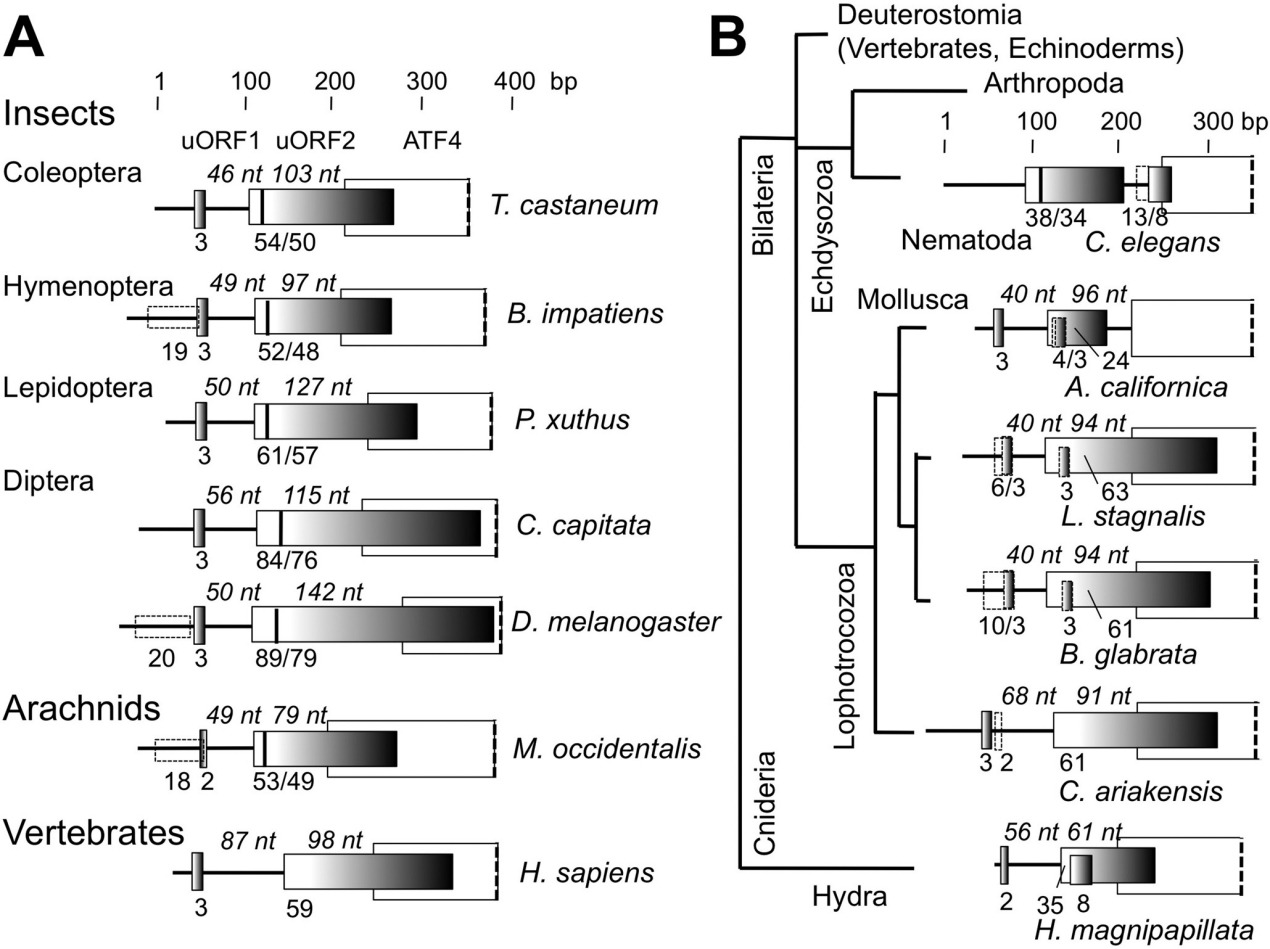
Conservation of the paired uORFs in ATF4 mRNA within metazoa. (A) ATF4 mRNA leader region from indicated arthropod species. Gray boxes indicate uORFs, with numbers below indicating their sizes in amino acids. A bar in the uORF2 denotes an in-frame AUG with a strong Kozak consensus. Numbers after a slash indicate the sizes of the smaller uORF starting with it. Open boxes indicate ATF4 ORFs. Dashed open boxes indicate an uORF starting with an AUG in a weak Kozak context. Italicized numbers designate the distance between the uORF1 stop codon and the uORF2 start codon or between start codons of uORF2 and ATF4. (B) Conservation of ATF4 uORF outside arthropods in metazoa. uORFs found in ATF4 leader regions of indicated species are depicted as in (A), except that we depicted a third short uORF overlapping with uORF2 that is often found in the groups of animals listed. Accession numbers for ATF4 mRNA sequences described in this figure are listed in Supplementary Information.

It is known that the mollusk *Aplysia* carries an ATF4 homolog with the same paired uORF arrangement (Figure 4B) as that for mammalian ATF4 (23) (Figure 4A, bottom). We also confirmed by cDNA sequencing that *T. castaneum* ATF4 possesses paired uORFs—a short, permissive uORF1 allowing downstream re-initiation, and a long, non-permissive uORF2 (Figure 4A) (see Supplementary text for details). Taking advantage of the homology established between *C. elegans* ATF-5, the *D. melanogaster* homolog, cryptocephal-A (Crc-A), and vertebrate ATF4 (24), we performed a thorough search for ATF4 homologs in metazoa and examined their 5′ UTR structure, if reported. As shown in Figure 4A, we found that all of the ATF4 homologs in insects with 5′ UTR information encode the paired uORFs. Interestingly, a second in-frame AUG start codon with a strong Kozak consensus is conserved within uORF2 of all insect ATF4 mRNAs (a thick vertical bar in Figure 4A). Moreover, this second start codon in uORF2 is conserved outside of the insect class (in a mite, which is an arachnid) within the arthropod phylum (Figure 4A). Another interesting feature is the presence of an uORF initiated by a start codon in poor Kozak context upstream of uORF1 (dashed boxes in Figure 4). Thus, in order for ATF4 regulation to operate, the start codon of this additional uORF must be bypassed through increased initiation accuracy or the ATF4 transcription must start after this start codon (see Discussion).

It is striking that the currently reported 5′ UTR of the ATF4 homolog (ATF-5) in *C. elegans* does not possess the typical uORF arrangement as found in mammals (25), even though the mollusk *Aplysia californica*, a metazoan species belonging to the superphylum lophotrocozoa (outside of echdysozoa containing arthropoda and nematoda) or *Hydra*, a cniderian, have one (Figure 4B). A short uORF1 encoding 2 or 3 amino acids is permissive to downstream re-initiation, because the ribosome that has translated uORF1 can be anchored to mRNA via an eIF (likely eIF3) and thereby resume scanning (26). However, the uORF1 of *C. elegans* ATF-5 mRNA appears to be too long, in order to allow for downstream re-initiation (see Discussion). As shown in Figure 1, nematoda is the only metazoan phylum, whose members lack 5MP. The co-conservation of 5MP and paired ATF4 uORFs suggests an intriguing possibility that 5MP in metazoa may have a specialized function in the regulation of ATF4 (see Discussion).

### ATF4 facilitates the expression of GADD34 eIF2α phosphatase in *T. castaneum*

Next, we proceeded to test whether 5MP is linked to the regulation of ATF4 by RNAi and expression studies by quantitative reverse transcriptase (qRT)-PCR. ATF4 governs amino acid synthesis and the oxidative stress response in mammalian cells (2) and promotes the growth of fibrosarcoma (27). Similarly, in *D. melanogaster*, the null mutation of Crc-A, the ATF4 homolog, causes early death before pupation (24). However, the transcriptional role of ATF4 in insects or any other metazoan species outside of mammals has not been studied. To fill this gap, we first wished to identify a transcriptional target of ATF4 by RNAi of ATF4 in *T. castaneum*. We focused on the phosphatase GADD34, which promotes dephosphorylation of eIF2α (28), a known direct downstream target of ATF4 in mammals (29,30). We chose *GADD34*, because ATF4 is typically regulated by eIF2α kinases and because the signal silencing by dephosphorylation is an important part of transcriptional regulation governed by many protein kinases (31).

**Figure 5.**
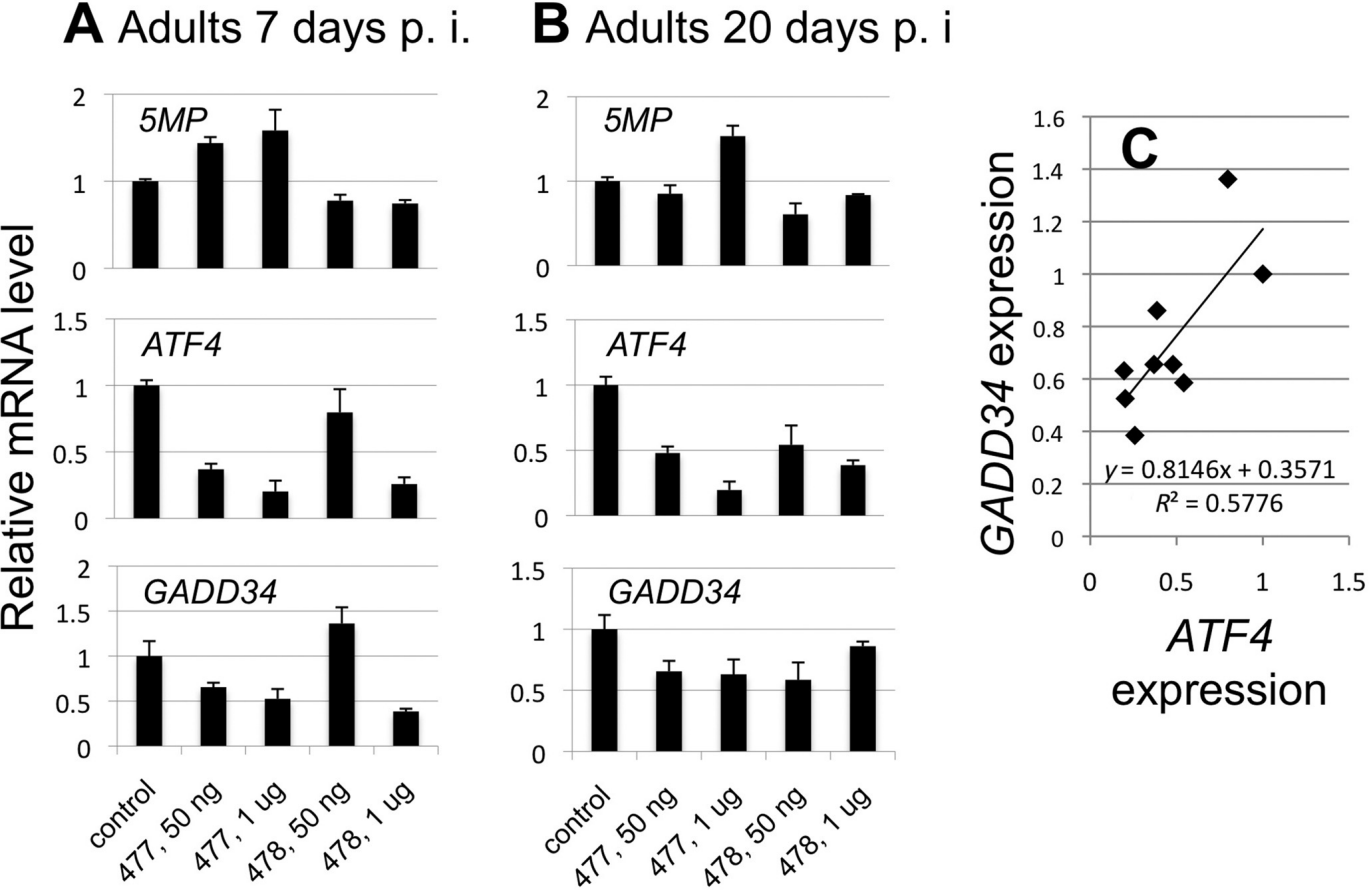
Effect of ATF4 RNAi on 5MP, ATF4 and GADD34 expression in *T. casteneum*. (A) and (B) RNA was isolated from adults 7 (panel A) or 20 days (panel B) after injection of 50 ng/μl (dashed lines) or 1 μg/μl (solid lines) of ATF4 dsRNA-477 or -478, ds RNA fragments corresponding to 5′- and 3′ half of ATF4 ORF, respectively. 5MP, ATF4, GADD34 and RPS3 mRNA levels were determined in triplicate in a single set of qRT-PCR (see Supplementary text) using gene-specific primers, as listed in Supplementary Table S1. The graphs show 5MP (top), ATF4 (middle) and GADD34 (bottom) mRNA levels relative to mock-treated control, after normalization by RPS3 data. Bars indicate SEM of the three technical replicates. (C) Correlation between *GADD34* and *ATF4* expression from data in (A) and (B).

Besides *GADD34*, we measured expression of *5MP* as well as *ATF4* as controls. For normalization, we used a well-characterized constitutively expressed mRNA, RPS3 (32). We knocked down *ATF4* with different doses of ATF4 RNAi and measured *5MP*,*ATF4*, and *GADD34* expression levels, relative to *RPS3*, in the RNAi-treated adults. We used two ∼500-bp double-stranded (ds) RNA fragments corresponding to non-overlapping portions of ATF4 ORF, termed ATF4 dsRNA-477 and -478, to exclude off-target effects coming from the region homologous to a portion of ATF4. As shown in Figure 5A and B, middle panels, we observed a dose-dependent decrease in ATF4 expression in adults treated with ATF4 dsRNA-477 and -478, respectively, both 7 and 20 days after injection. *GADD34* expression was reduced in all cases (Figure 5A and B, bottom panels), except on day 7 after a low dose of dsRNA-478 was injected and only a minimal effect on *ATF4* expression was observed (Figure 5A, middle panel, fourth column). This last exceptional data reinforce that the specific effect of ATF4 RNAi, but not the amount of dsRNA itself, determines the level of *GADD34* expression (compare to other experiments using 50 ng of ATF4 dsRNA in Figure 5A or B, middle panels). In further support of the specific effect, *5MP* expression was not reduced significantly by RNAi treatment (Figure 5A and B, top panels). Importantly, the effect of ATF4 RNAi treatment on *GADD34* was always smaller than that on *ATF4* (*P* = 0.002, *n* = 8). The plot in Figure 5C suggests that the degree of decrease in *ATF4* expression caused by ATF4 RNAi correlates well with a decrease in *GADD34* expression, and that 30–40% of *GADD34* expression is ATF4-independent. These data confirm that *GADD34* is a downstream target of ATF4 in insects, similar to *GADD34* in mammals.

### Beetle 5MP facilitates the expression of *GADD34* without increasing ATF4 mRNA abundance

**Figure 6.**
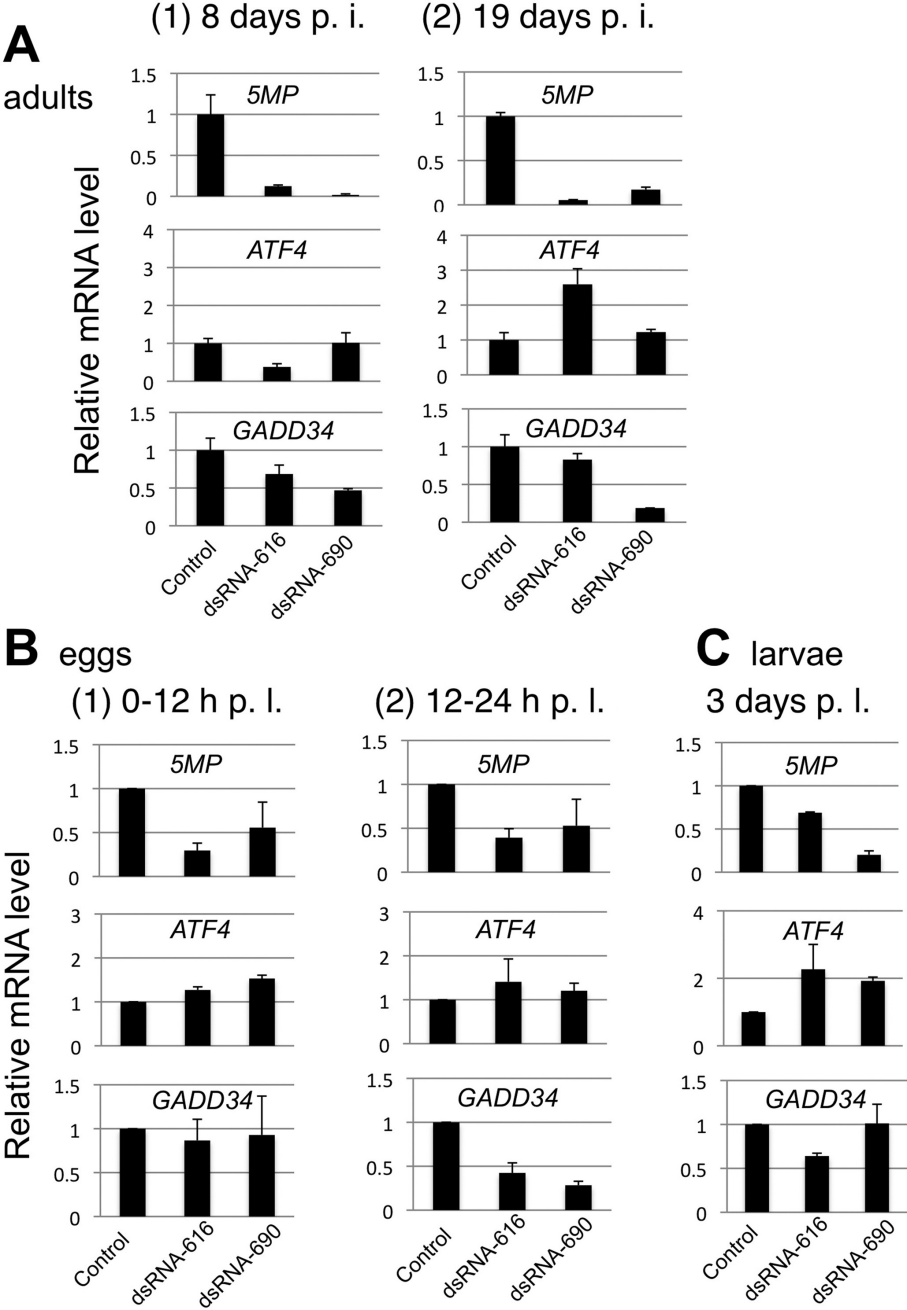
Effect of 5MP RNAi on expression of 5MP, ATF4 and GADD34 in *T. castaneum*. (A) Relative mRNA expression for 5MP (top), ATF4 (middle), and GADD34 (bottom) in beetles 8 (panel 1) and 19 (panel 2) days post-injection (p. i.) of two non-overlapping RNAi fragments (1 μg/μl) directed to 5MP, termed 5MP dsRNA-616 or dsRNA-690. RNA abundance was quantified with qRT-PCR and presented as in Figure 5A and B. (B) Expression in eggs 0–12 h (panel 1) or 12–24 h (panel 2) post-lay (p. l.) by injected adults. In panel 1, 5MP, ATF4 and GADD34 expression normalized to RPS3 was examined right after the injected adults were allowed to lay eggs for 12 h and presented in values relative to expression from mock-treated adults. In panel 2, expression was similarly examined after the eggs collected similarly were aged for an additional 12 h. (C) 5MP, ATF4 and GADD34 expression normalized to RPS3 in larvae 3 days post-lay by injected adults. Presented are values relative to expression from mock-treated adults. In (B) and (C), the average of two biological replicates are shown with SEM.

Having shown that GADD34 is a target of ATF4, we examined the effect of 5MP knockdown on *GADD34* and *ATF4* expression. As shown in Figure 6A, top panels, qRT (reverse transcriptase)-PCR analysis indicated that treatment with two non-overlapping dsRNA fragments directed against the 5MP-coding region, 5MP dsRNA-616 and -690, substantially reduced 5MP mRNA expression compared to control treatment in 8 and 19 days after ds RNA injection. Importantly, we found that 5MP dsRNA-690 treatment reduced *GADD34* expression without altering ATF4 mRNA levels (Figure 6A, middle and bottom panels, columns 3). Although 5MP dsRNA-616 altered ATF4 mRNA levels, it also reduced *GADD34* expression (Figure 6A, columns 2). Together with data from two more sets of injected adults (for 8 days), 5MP RNAi treatment significantly reduced *GADD34* expression (*P* = 0.003, *n* = 6). On average from the six experiments, *GADD34* expression was reduced to 51% (with SEM 8%) at the whole body level. Since *GADD34* expression levels would vary widely between different tissues, the significant, 2-fold decrease in GADD34 mRNA level, caused by 5MP RNAi treatment, is impressive, and strongly suggests that 5MP facilitates *GADD34* expression.

In search of additional evidence that 5MP facilitates *GADD34* expression, we examined mRNA expression in eggs that dsRNA-injected beetles laid or larvae that hatched from them. As shown in Figure 6B and C, top graphs, the knockdown effect on *5MP* expression was apparent in eggs and continued through newly hatched larvae, although the effect was clearly attenuated. Importantly, 5MP RNAi treatment significantly reduced *GADD34* expression (*P* = 0.04, *n* = 4) without altering ATF4 mRNA abundance in eggs that developed for 12–24 h after being laid (Figure 6B, panel 2, middle and bottom graphs). 5MP RNAi treatment generally increased ATF4 mRNA expression in eggs or larvae (Figure 6B and C, middle graphs), suggesting a compensatory effect caused by potential reduction in ATF4 activity. These results support the model that 5MP facilitates *ATF4* expression mainly through a translational mechanism, ultimately up-regulating *GADD34* transcription.

### 5MP is essential for development of *T. castaneum*

**Figure 7.**
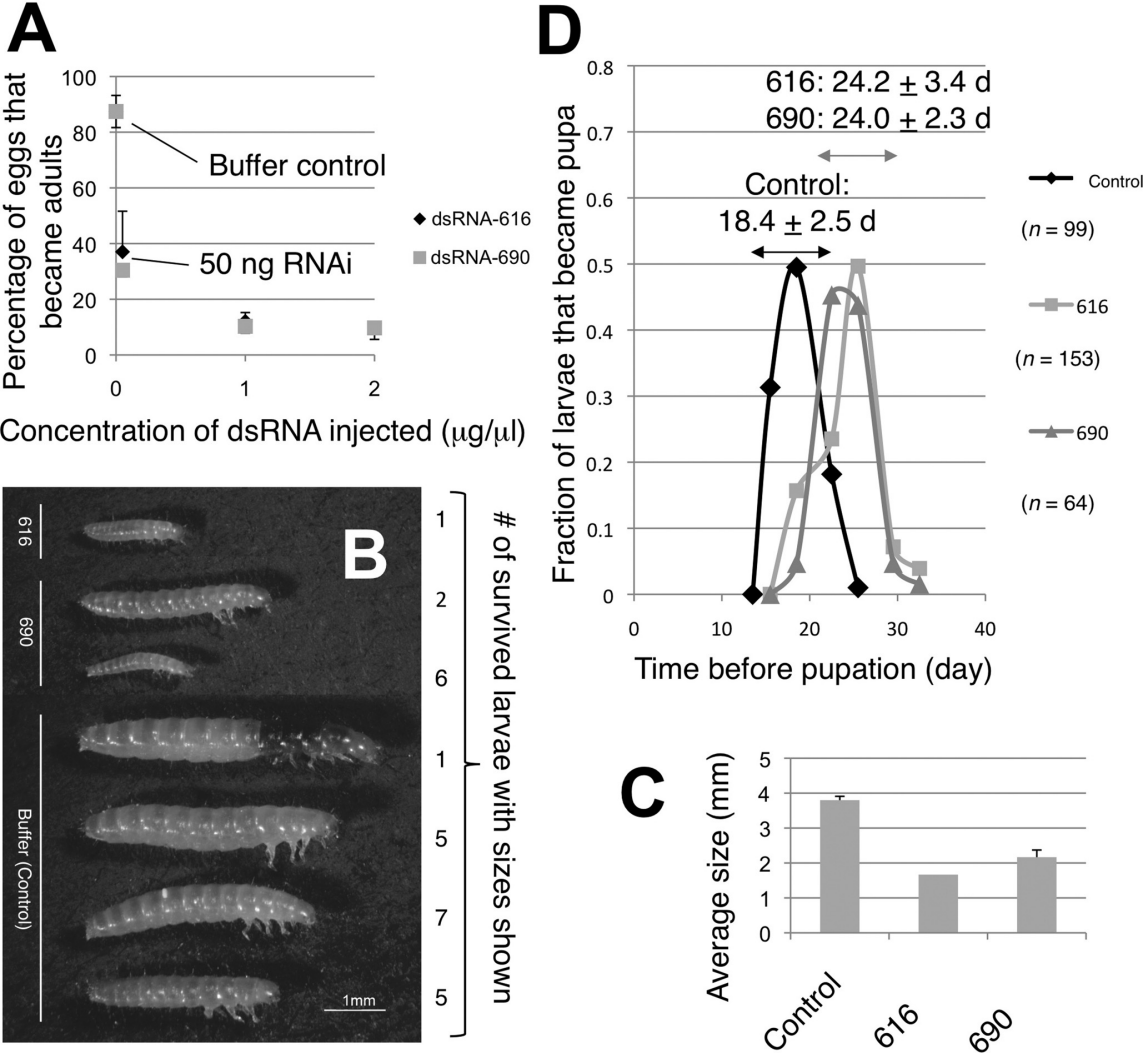
Effect of 5MP RNAi on the development of *T. castaneum*. (A) Titration experiment. A group of adults was injected with indicated concentrations of 5MP dsRNA-616 (black diamonds) or dsRNA-690 (gray triangles) and allowed to lay eggs to assess the effect of RNAi on development of the progenies (see Supplementary file). Graph indicates adult survival rates by each treatment with bars indicating SEM (*n* = 3–7). In (B–D), dsRNA was injected at 1 μg/μl. In (B), larvae were taken out from a jar initially containing 25 eggs after 14 days of egg collection and photographed. Graph in (C) shows the average size of the population in the jar. In (D), the time to become pupa was calculated for all examined individuals from the mid-point between the day the pupa was observed and the last day of larva observation since the day of egg collection. The average time to become pupa and SD are indicated for the group of larvae under each treatment.

In *D. melanogaster*, the genes coding for 5MP (10,11) and ATF4/Crc-A (24) are essential for larval development, which supports the presence of a genetic link between 5MP and ATF4 regulation. To examine whether 5MP is essential in *T. castaneum*, we injected different doses of 5MP dsRNA-616 and 5MP dsRNA-690 to fecund female beetles, and examined the development of insects from eggs laid by them. Adult survival, egg laying and egg hatch rates were similar to control treatments. However, only ∼30% or 10% of the larvae grew to adults from parents injected with 50 ng/μl or 1 μg/μl dsRNA of either, respectively (Figure 7A). The dose-dependent response appeared to reach a plateau at 2 μg/μl 5MP dsRNA-690 as ∼10% of the larvae from beetles injected with this amount grew to adults also (Figure 7A). The surviving larvae from RNAi-treated parents are, in general, smaller than those from the mock-treated parents, but look identical compared to normal larvae from mock-treated parents (Figure 7B and C). Furthermore, it took, on average, 6 days longer for them to pupate (Figure 7D, see Supplementary Figure S2 for a smaller-scale repeat with new injections). These results indicate a spectrum of RNAi-effects from premature death at the larval stage (∼90%) to a slow-growing and extended larval period (∼10%). Since the few that developed into pupae continued to develop to adults, we conclude that 5MP is essential for the growth of *T. castaneum* during the larval stage.

Having observed these severe growth defects, we more closely examined the developing embryos. However, we did not observe any morphological defects caused by RNAi treatment. Additionally, we stained the embryos with anti-horseradish peroxidase, but did not observe any changes in neuronal growth. *In situ* hybridization of embryos from untreated parents indicated that 5MP mRNA is expressed throughout the body of the embryos. Thus, 5MP appears to be a housekeeping in *T. castaneum*. However, its higher abundance in neurons has not been ruled out (see Discussion).

In contrast to the strong effect on larval development caused by 5MP RNAi, we observed only a minor effect of ATF4 RNAi on the development of *T. castaneum*. This might be expected from weaker effects of ATF4 RNAi on *ATF4* expression (Figure 5A and B, middle graphs) than effects on 5MP expression observed by 5MP RNAi (Figure 6A, top graphs), using the same amount (1 μg/μl) of dsRNA. However, we observed that the larvae that hatched from eggs collected early after ATF4 RNAi injection pupated, on average, ∼1 day later than those from mock-treated beetles (Supplementary Figure S3A and B, *P* = 0.03, *n* = 4). We also found a few outliers in larvae from ATF4 RNAi-treated beetles, which pupated 27 days or more after egg lay or lived longer than 27 days but died before 35 days (Supplementary Figure S3C). Note that *all* the larvae from control beetles pupate between 12 and 25 days (Figure 7D, E2 and E3C, row 1). Therefore, we conclude that the effect of ATF4 RNAi penetrated to a minority (1∼3%) of progenies, displaying the defect in larval development, as observed with beetles treated with 5MP RNAi (Figure 7D) and flies deleted for 5MP and ATF4 (10,24). Together, these data endorse the genetic link between 5MP and ATF4 regulation during larval development in *T. castaneum*, which was observed in *D. melanogaster*.

## DISCUSSION

In this work, we conducted a thorough survey of 5MP homologs from eukaryotes and found that this protein is highly conserved and maintained under purifying selection, even though they are missing in two major phyla, nematoda and ascomycota (Figure 1). The phylogenetic tree also supports the notion that this protein existed early in eukaryotic evolution before the major divergence into plant and animal/fungi kingdoms, as proposed previously (8). Another interesting finding from our phylogenetic analysis is that 5MP gene duplications occurred independently in plant, chordate and *Drosophila* lineages (Figure 1). Maintenance of copies in multiple lineages indicates different roles or expression patterns of the duplicated 5MP homologs.

### 5MP counteracts the activity of eIF5

eIF5 is made of two domains, N-terminal GTPase activating protein domain and C-terminal α-helical domain termed W2. However, the NTD of 5MP is unrelated to eIF5-NTD and does not possess the GAP activity. 5MP is therefore a competitive inhibitor of eIF5 (5). Our heterologous expression study in the yeast system confirmed the conserved role of 5MP across the Eukaryal domain of life in translational inhibition through binding eIF2 (Figure 2). We previously characterized human 5MP1 in yeast in a greater detail (5), showing that 5MP inhibits the recruitment and recycling of eIF2 through inhibiting its association with eIF5. Since the effects of the expressed 5MP homologs were quantitatively similar to that of human 5MP1 (Figure 2F), our results reinforce that the 5MP homologs from diverse eukaryotes control translation by the mechanisms similar to human 5MP1. Human 5MP1 binds human eIF2β (Figure 3) at an affinity similar to that reported for human eIF5 binding to eIF2β (16), confirming that 5MP can serve as a competitor with eIF5.

We previously showed that human 5MP1 inhibits translation in rabbit reticulocyte lysates and reduces polyribosome abundance in HeLa cells (5). On the contrary to the idea that 5MP is a translation inhibitor, however, 5MP expression does not retard yeast growth or produce a strong induction of general control response (Figure 2E and F), even though a substantial fraction of eIF2 is bound to 5MP (Figure 2C). Recently, the guanine nucleotide exchange factor eIF2B was reported to possess an activity to dissociate eIF5 from eIF2:GDP (33). It is possible that eIF2B works to maintain active translation through dissociating inhibitory eIF2 complexes not only with eIF5 but also with 5MP. The weak induction of general control response by 5MP may be explained by the idea that 5MP inhibits translation initiation at uORF1 or *GCN4* start codon. Because strong eIF2-eIF5 contact on the ribosome is required for stabilizing initiating ribosomes at AUG codons (16,34–36), 5MP can compete with this contact (Figure 3), thereby inhibiting the initiating ribosome. Therefore, even though 5MP may retard the TC recruitment to the ribosome migrating *GCN4* mRNA leader region, *GCN4* translation might not be induced, as observed for some of the eIF5-CTD mutations (37).

### Does 5MP control the expression of ATF4?

Here we report that 5MP is essential for larval development, using the red flour beetle, *T. castaneum*, as a model (Figure 7). Based on similar lethal phenotypes caused by ATF4 and 5MP deletions in the fruit fly (10–11,24), we hypothesize that the essential role of 5MP is to facilitate translation of ATF4, the transcription factor responsible for stress resistance. We presented evidence that 5MP facilitates the expression of *GADD34*, a known transcriptional target of ATF4 (Figures 5 and 6), which is consistent with this model.

The potential co-conservation of 5MP and the paired uORFs found in ATF4 leader region (Figure 4) warrants further studies on the mechanism of ATF4 translational control across metazoa. Based on the current genome database, the *C. elegans* ATF-5 has two uORFs (Figure 4B), but its uORF1 is too long to be permissive for downstream re-initiation (26). The ability of the ribosome to resume scanning after uORF translation depends on initiation factors (such as eIF3 or eIF4G) staying bound to the ribosome after the completion of the uORF translation. An uORF longer than 20 amino acids does not normally permit re-initiation, which is likely due to the lack of these required factors (26). Since GCN2 eIF2α kinase homolog promotes stress-induced ATF-5 translation in *C. elegans* (25), it will be crucial to determine the ATF-5 mRNA structure in *C. elegans* and understand the mechanism by which *ATF-5* translation is induced by eIF2 inhibition.

While 5MP may facilitate ATF4 translation by inhibiting eIF2 in tissues with high expression of 5MP, the ability of 5MP to bind eIF3—as demonstrated *in vitro* (5) and observed in yeast (Figure 2B and C)—suggests that 5MP can also act on the ribosome even at its normal abundance. For example, it is possible that 5MP facilitates re-initiation after uORF1 translation by assisting the linkage of the required eIFs to the ribosome (also see below). Alternatively, 5MP may still work outside of the ribosome, for example, in order to increase a regulatory response through eIF2 phosphorylation by not having all of the eIF2 in the active form.

Our survey of *ATF4* 5’ UTR also revealed potentially new features of *ATF4* control through uORFs in arthropods (Figure 4), a second in-frame start codon in uORF2, and an additional uORF upstream of uORF1, initiated by a start codon in a poor Kozak context. The former is expected to lower the basal *ATF4* translation in the absence of inducing signals, thereby increasing the magnitude of regulation caused by the input of signals. However, one question remains how the additional upstream uORF can be bypassed for the appropriate control of *ATF4* through uORFs. As mentioned above, 5MP binding to the ribosome may inhibit translation initiation through direct competition with the critical eIF5-eIF2 contact. If this is the case, 5MP expression may increase *ATF4* translation through increasing the initiation accuracy and inhibiting translation of the additional uORFs starting with a start codon in a poor context (Figure 4). To examine the variety of possible mechanisms by which 5MP controls *ATF4* in the entire metazoa, more *ATF4* expression studies in insect cells, as done previously for mammalian *ATF4* control (3), are necessary. Recently established cultured cell lines from *T. castaneum* will facilitate such studies (38).

### The role of 5MP in higher-ordered function of animals

In the fruit fly, *Drosophila melanogaster*, 5MP is implicated in memory (9) and axon guidance (10). Axon guidance is governed by Slit/Robo signaling. Recently, RNAi knockdown of *Tca* Robo was shown to cause ectopic midline crossing of longitudinal axons (39), similar to the phenotypes observed with null mutants of *robo* or *kra/5MP* in *D. melanogaster* (10). As null mutations in *robo* and other axon guidance genes often display embryonic or larval lethality, an axon guidance role for *T. castaneum* 5MP might help to explain the lethality we observe with 5MP RNAi (Figure 7). Interestingly, in mammals, ATF4 is an inhibitor of synaptic plasticity and memory (40). These findings warrant the further study on relationship between 5MP and ATF4 expression in the higher-ordered function of animals, using insect as the model organism.

## SUPPLEMENTARY DATA

Supplementary Data are available at NAR Online.

SUPPLEMENTARY DATA
