# Supplementary material for: Essential role of eIF5-mimic protein in animal development is linked to control of ATF4 expression
Source: Nucleic Acids Res. 2014 Aug 21;42(16):10321–30. doi: 10.1093/nar/gku670 (PMC4176352; doi:10.1093/nar/gku670)
Supplement: SUPPLEMENTARY DATA [file supp_42_16_10321__index.html]

Essential role of eIF5-mimic protein in animal development is linked to control of ATF4 expression — Essential role of eIF5-mimic protein in animal development is linked to control of ATF4 expression — SUPPLEMENTARY DATA 

# Essential role of eIF5-mimic protein in animal development is linked to control of ATF4 expression

## SUPPLEMENTARY DATA

**Files in this Data Supplement:**

- SUPPLEMENTARY DATA
- SUPPLEMENTARY DATA
