# Supplementary material for: Essential role of eIF5-mimic protein in animal development is linked to control of ATF4 expression
Source: Nucleic Acids Res. 2014 Aug 21;42(16):10321–30. doi: 10.1093/nar/gku670 (PMC4176352; doi:10.1093/nar/gku670)
Supplement: SUPPLEMENTARY DATA [file supp_gku670_nar-00820-v-2014-File010.pdf]

Hiraishi_Fig. S1

A

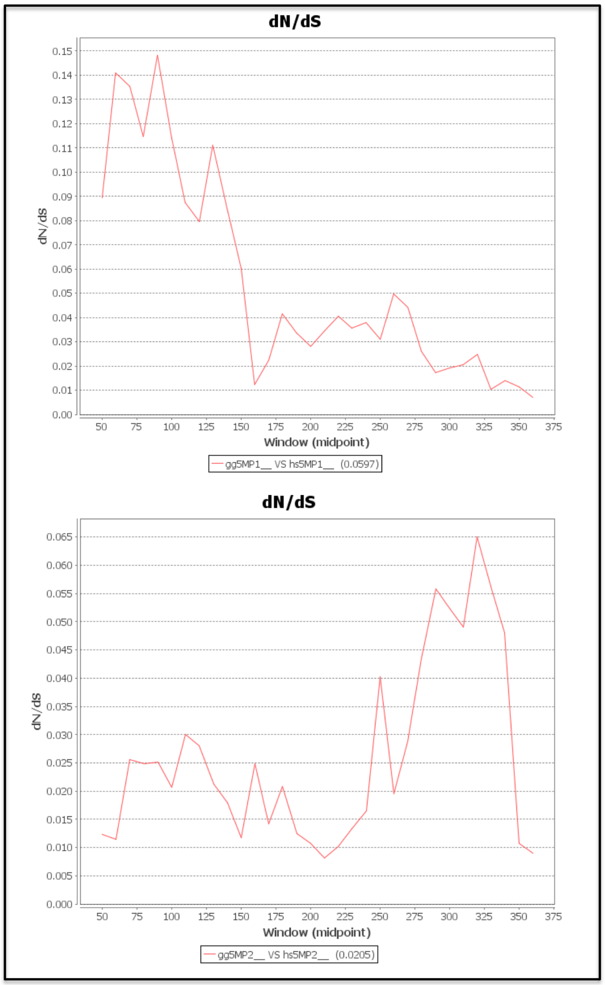

B

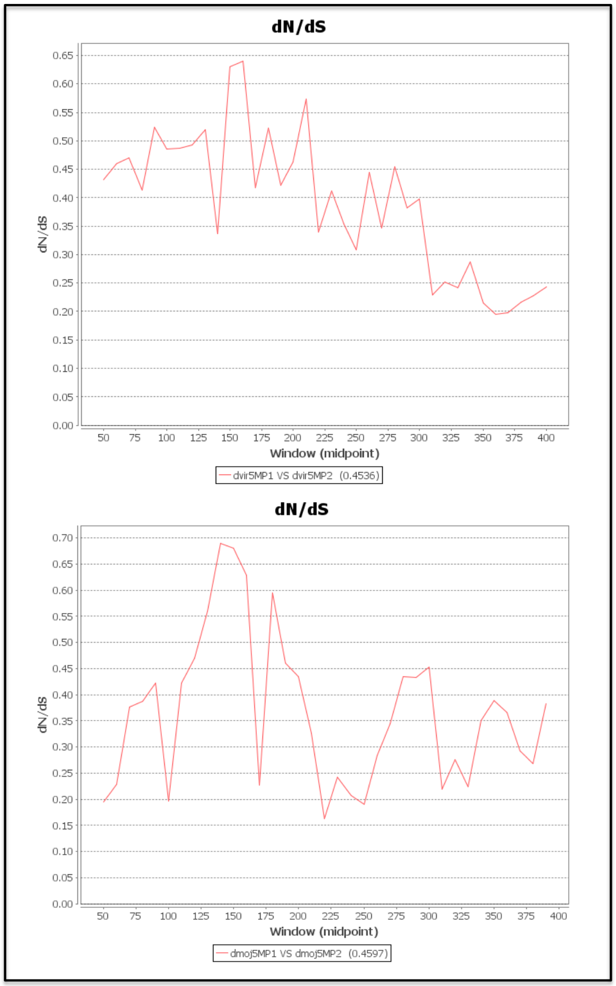

Hiraishi_Fig. S2

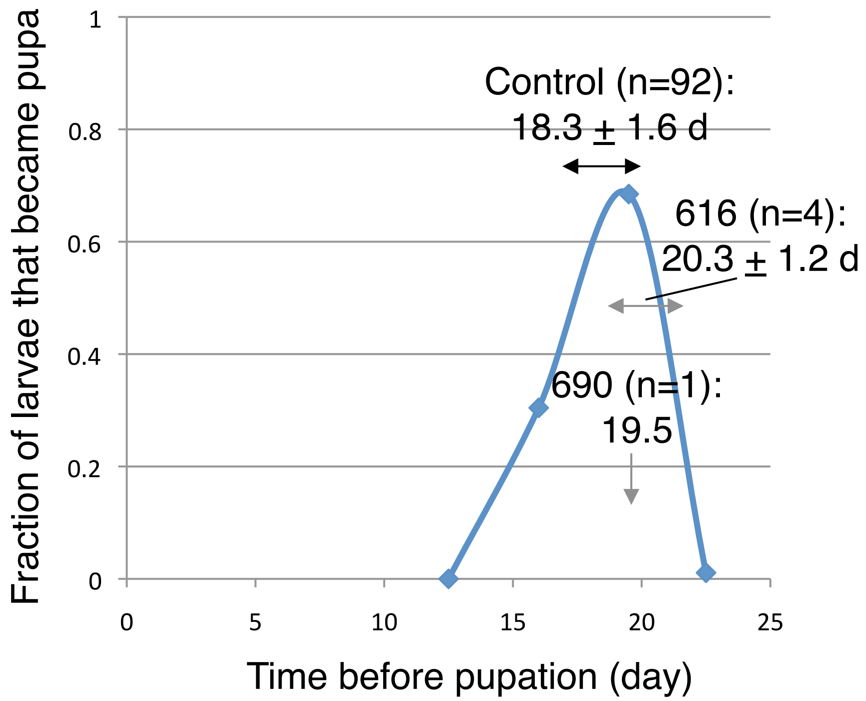

Hiraishi_Fig. S3

A Larvae that hatched from the first egg collection (3 days after dsRNA injection)

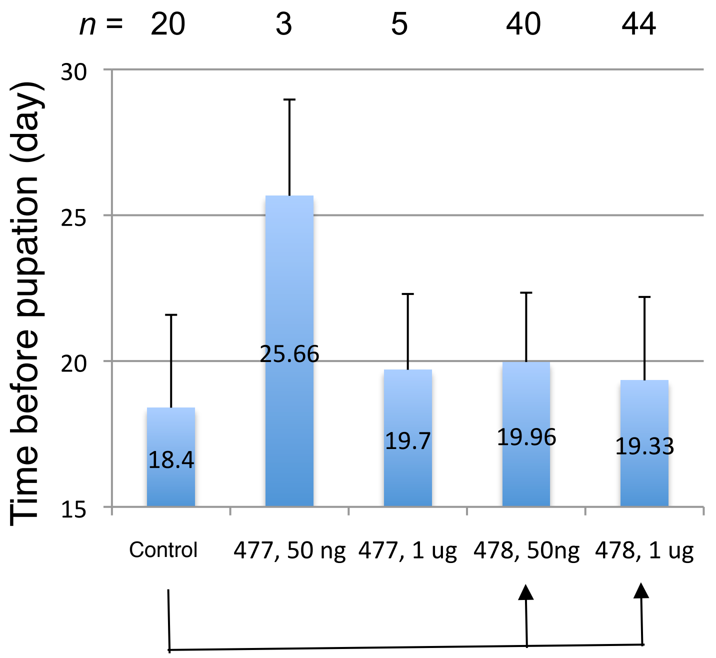

B Larvae that hatched from the *first two* egg collections (3 and 6 days after dsRNA injection)

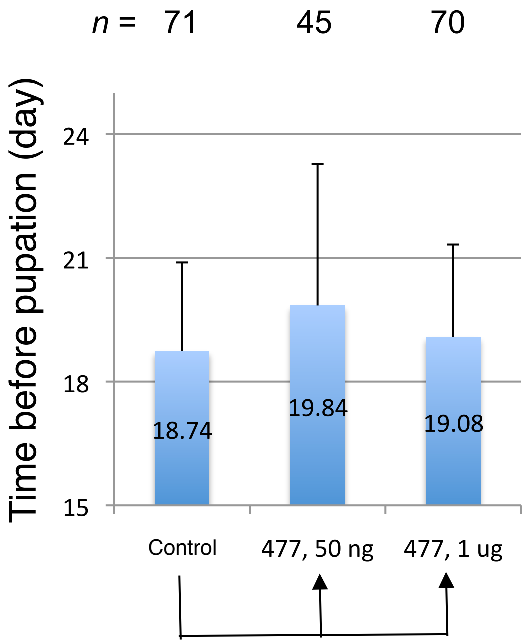

p = 0.03 (n = 4)

C

# of Larvae	Pupated between 12 and 25 days	Pupated between 27 and 35 days	Lived for >27 days but died before 38 days
1. Control	128 (100%)	0 (0 %)	0
2. dsRNA-477, 50 ng	142 (96.6%)	5 (3.4%)	0
3. dsRNA-477, 1 μg	134 (98.5%)	2 (1.5%)	1
4. dsRNA-478, 50 ng	166 (100%)	0 (0%)	0
5. dsRNA-478, 1 μg	165 (98.8%)	1 (0.6%)	5
