# Supplementary material for: Essential role of eIF5-mimic protein in animal development is linked to control of ATF4 expression
Source: Nucleic Acids Res. 2014 Aug 21;42(16):10321–30. doi: 10.1093/nar/gku670 (PMC4176352; doi:10.1093/nar/gku670)
Supplement: SUPPLEMENTARY DATA [file supp_gku670_nar-00820-v-2014-File009.doc]

**Supplementary file for Hiraishi et al.**

**Phylogenetic analyses**

We conducted a comprehensive database analysis using various 5MP protein sequences as probes in order to survey the occurrence (presence) of 5MP homologs. The protein sequences of 5MP homologs from eukaryotes were identified in BLAST and PSI-BLAST searches using previously identified 5MP homologs (1), (2). Database searches for 5MP homologs from selected groups of eukaryotes were performed by restricting the searches for those groups (e.g. nematodes). Among protozoans, a 5MP homolog was only identified in three strains of *G. intestinalis*. 5MP homologs were identified in many species belonging to the viridiplantae, fungi and metazoa kingdoms, with many species containing more than one 5MP homolog. Notable exceptions of phyla in which we did not identify 5MP homologs are nematodes and Ascomycota fungi.

97 5MP protein sequences used in the phylogenic analysis are listed in the next section. These protein sequences were aligned using MUSCLE, a multiple sequence alignment software (3), and the resulting alignment was manually edited using MacClade (4) by removing the N-terminal and C-terminal ends that do not align well and are of variable length. The phylogenetic analysis from this dataset was performed using a Maximum Likelihood approach (5) with nodal support assessed via bootstrapping (100 pseudo-replicates) (6), as implemented in PhyML 3.0 (7). For the resulting alignment, the best-fit model of amino acid replacement was selected using ProtTest 2.4 (8). The model chosen for this analysis was LG+I+G. The phylogenetic tree was rooted using three different strains of *Giardia intestinalis* as outgroups.

During the course of the analysis, we found that 5MP gene duplications occurred independently in plant, chordate and *Drosophila* lineages. Accordingly, we arbitrarily assigned numbers (1 and 2) and letters (a and b for fish 5MP) for 5MP copies. The same numbers only reflect a close relationship for 5MP genes from closely related species, e.g. for vertebrate 5MP copies, but not for 5MP homologs of distantly related organisms, such as plant vs. vertebrate sequences. Two 5MP copies are found in three plant species (*P. patens*, *A. thaliana* and *Z. mays*), which originated from three independent and relatively recent duplication events. The only arthropod species with two 5MP copies are *D. virilis* and *D. mojavensis*, which both belong to the subgenus *Drosophila*, whereas fruit fly species that belong to other subgenera contain only one 5MP copy. Since the basal branches in the insect 5MP clade received only low bootstrap support, the origin of the second copy in *D. virilis* and *D. mojavensis* is currently unclear. However, the long branch-lengths of the 5MP-2 copies indicate rapid diversification after the initial duplication event. Multiple 5MP copies are also found in chordates. Two closely related copies were identified in the lancelet *B. floridae*, which share 83% amino acid identity and likely originated from a gene duplication event in that lineage. A third partial *B. floridae* 5MP copy (XP_002590958) was found in database searches, which lacks the first 28 N-terminal amino acids and was thus excluded from the phylogenetic analysis. Among all available 5MP sequences, this copy is most closely related to *B. floridae* 5MP-2 and 5MP-1, displaying 87% and 79% amino acid identity, respectively.

Two main 5MP clades were formed by vertebrate 5MP proteins: one that contains *D. rerio* and tetrapod 5MP1 and a second clade that contains both vertebrate and fish 5MP2, suggesting an early duplication in the vertebrate ancestor. All fish species surveyed in this study contain two 5MP2 copies, which were designated as 5MP2a and 5MP2b and which likely originated from a duplication in a common ancestor of the surveyed fish species. Interestingly, *D. rerio* is the only fish species for which an ortholog of tetrapod 5MP1 was identified in the protein database. However, partial putative 5MP1 orthologs were identified in other *Cypriniformes* fish species such as *Pimephales promelas* (fathead minnow) and *Rutilus rutilus* (roach minnow) in expressed sequence tag (EST) database searches using TBLASTN (data not shown). Thus it appears that the 5MP2 copy was duplicated in an early ancestor of teleost fish and that the 5MP1 copy got subsequently lost in some fish lineages.

**5MP protein sequences used in the phylogenetic analysis**

Genbank accession numbers for 5MP protein sequences are (from top of Fig. 1): *Giardia intestinalis* ATCC 50581, EET01726; *Giardia intestinalis* ATCC 50803 (strain WB), XP_001706394; *Giardia intestinalis* P15, EFO65243; *Chlamydomonas reinhardtii,* XP_001691456; *Volvox carteri,* XP_002949287; *Chlorella variabilis*, EFN59008; *Coccomyxa subellipsoidea*, EIE25009; *Selaginella moellendorffii,* XP_002992407; *Physcomitrella patens* 5MP-1, XP_001758587; *Physcomitrella patens* 5MP-2, XP_001785917; *Ricinus communis,* XP_002514839; *Arabidopsis thaliana* 5MP-1, NP_564845; *Arabidopsis thaliana* 5MP-2, NP_568534; *Triticum aestivum*, AFW20017; *Zea mays* 5MP-1, NP_001132772; *Sorghum bicolor*, XP_002489171; *Zea mays* 5MP-2, NP_001150633; *Chondrus crispus*, CDF36343; *Puccinia triticina*, PTTG_05472.4 (Broad Institute); *Cryptococcus neoformans*, XP_775830; *Postia placenta*, XP_002469360; *Schizophyllum commune*, XP_003029464; *Laccaria bicolor*, XP_001877720; *Coprinopsis cinerea*, XP_001830176; *Monosiga brevicollis*, XP_001744944; *Hydra magnipapillata*, XP_002156389; *Capitella teleta*, ELT97012; *Crassostrea gigas*, EKC29192; *Aplysia californica*, XP_005100739; *Strongylocentrotus purpuratus*, XP_792300; *Branchiostoma floridae* 5MP-1, XP_002612929; *Branchiostoma floridae* 5MP-2, XP_002612932; *Danio rerio* 5MP-1, NP_957212; *Xenopus tropicalis* 5MP-1, XP_002933339; *Homo sapiens* 5MP-1, Q9Y6E2; *Anolis carolinensis* 5MP-1, XP_003222184; *Chrysemys picta bellii* 5MP-1, XP_005306711; *Taeniopygia guttata* 5MP-1, XP_002188448; *Gallus gallus* 5MP-1, Q5ZL42; *Melopsittacus undulates* 5MP-1, XP_005152932; *Xenopus tropicalis* 5MP-2, Q6P2Z0; *Anolis carolinensis* 5MP-2, XP_003215217; *Chrysemys picta bellii* 5MP-2, XP_005310368; *Homo sapiens* 5MP-2, Q7L1Q6; *Gallus gallus* 5MP-2, Q5ZLT7; *Melopsittacus undulates* 5MP-2, XP_005145607; *Taeniopygia guttata* 5MP-2, XP_002190923; *Danio rerio* 5MP-2b, NP_998257; *Maylandia zebra* 5MP-2b, XP_004563379; *Oreochromis niloticus* 5MP-2b, XP_005452376; *Oryzias latipes* 5MP-2b, XP_004066439; *Takifugu rubripes* 5MP-2b, XP_003966731; *Tetraodon nigroviridis* 5MP-2b, CAG10296; *Danio rerio* 5MP-2a, NP_956002; *Maylandia zebra* 5MP-2a, XP_004566089; *Oreochromis niloticus* 5MP-2a, XP_003443375; *Oryzias latipes* 5MP-2a, XP_004082100; *Takifugu rubripes* 5MP-2a, XP_003962113; *Tetraodon nigroviridis* 5MP-2a, CAG10097; *Metaseiulus occidentalis*, XP_003748616; *Ixodes scapularis*, XP_002434413; *Amblyomma variegatum*, DAA34728; *Caligus clemensi*, ACO14947; *Lepeophtheirus salmonis*, ACO12029; *Caligus rogercresseyi*, ACO10382; *Daphnia pulex*, EFX77858; *Acyrthosiphon pisum*, XP_003243818; *Riptortus pedestris*, BAN20563; *Pediculus humanus corporis*, XP_002429739; *Tribolium castaneum*, XP_973636; *Dendroctonus ponderosae*, AEE63437; *Bombyx mori*, NP_001091797; *Helicoverpa armigera*, ACD74811; *Danaus plexippus*, EHJ74619; *Nasonia vitripennis*, XP_001608265; *Camponotus floridanus*, EFN70895; *Harpegnathos saltator*, EFN88555; *Megachile rotundata*, XP_003701374; *Bombus terrestris*, XP_003401425; *Bombus impatiens*, XP_003494274; *Apis mellifera*, XP_395256; *Apis florea*, XP_003695908; *Anopheles gambiae*, XP_312539; *Aedes aegypti*, XP_001655819; *Culex quinquefasciatus*, XP_001849124; *Drosophila virilis* 5MP-2, XP_002057137; *Drosophila mojavensis* 5MP-2, XP_002010950; *Ceratitis capitata*, XP_004518195; *Drosophila willistoni*, XP_002072079; *Drosophila grimshawi*, XP_001994692; *Drosophila mojavensis* 5MP-1, XP_001998356; *Drosophila virilis* 5MP-1, XP_002053650; *Drosophila persimilis*, XP_002017410; *Drosophila pseudoobscura*, XP_001359528; *Drosophila ananassae*, XP_001967515; *Drosophila melanogaster*, NP_524238; *Drosophila sechellia*, XP_002038438; *Drosophila erecta*, XP_001978954; *Drosophila yakuba*, XP_002098038.

**Cloning and expression of 5MP cDNA**

As listed in Table S1, the 5’(MluI)- and 3’(BamHI or BglII)- specific primers for 5MP coding regions from *G. intestinalis (Gin)*, *T. castaneum* (*Tca*, red flour beetle), *T. aestivum* (*Tae*, wheat), and *P. triticina* (*Ptr*, fungal pathogen) were designed based on the DNA sequences that were deposited in Genbank (*Gin*, XM_001706342; *Tca*, XP_973636; *Ptr*, Broad Institute PTTG_05472.1), or a *Tae* 5MP sequence that we generated from 8 EST database (Genbank Acc # TPA_BK008647).

Next, DNA fragments coding for *Gin*, *Tca*, *Tae*, and *Ptr* 5MP homologs were PCR-amplified (Promega GoTaq^TM^ DNA polymerase) using the 5’- and 3’- specific primers and cDNAs prepared as follows: The *Gin* and *Tca* cDNA templates were prepared with Superscript^TM^ III First-strand synthesis system (Invitrogen) using *Gin* RNA that was isolated from the strain WB (ATCC50803) (a gift of Dr. C-C Wang, UCSF)*,* and *Tca* RNA that was isolated from pupae using RNeasy Mini kit (Qiagen). To get *Tca* RNA, several pupae were quickly frozen in a microcentrifuge tube in liquid nitrogen and thawed immediately at room temperature. Pupae were homogenized with a plastic pestle and then suspended in 350 μl RLT buffer containing 1% β-mercaptoethanol. The lysate was passed through a 20-gauge needle 10-20 times and the debris removed by microcentrifugation at 12,000 rpm for 3 min. The cleared lysate was immediately mixed with freshly prepared 70% ethanol and loaded onto the spin column.

Urediniospores of *P. triticina* race MHDS were inoculated onto seedlings of the wheat cultivar Thatcher. Seedlings were at the 2-3 leaf seedling stage. Urediniospores were suspended in Soltrol 170 (Conoco-Phillips Petrolium, Bartelsville, OK) and applied to leaves using an atomizer at 40 PSI. Seedlings were placed in a mist chamber for 16 h at 100% humidity and 20^o^C. Seedlings were then placed in a controlled environment chamber at 18^o^C and 16 h:8 h day:night cycles. At 6 days post inoculation, which is one day prior to spore eruption, leaf tissue was harvested and quickly frozen in liquid nitrogen. Heavily infected tissue was used to isolate total RNA using the mirVana RNA kit (Life Technologies, CA). cDNA was constructed using 500 ng of total RNA, oligo d(T)_18_, and Superscript RT II, according to suggested protocol (Life Technologies). This cDNA was used to obtain both *Ptr* and *Tae* 5MP coding DNAs.

The PCR products were cloned into the pGEM-T easy vector (Promega). Several T-vector clones were isolated for each of *Gin, Tca, Tae* and *Ptr* 5MP and sequenced using primers flanking the cloning sites. *Gin* 5MP DNA sequence (KC196541) was identical to the original sequence from the WB strain (XM_001706342). *Tca, Tae*, and *Ptr* 5MP sequences had many base substitutions compared to the references, which sometimes altered amino acid sequences (Table S2-S4; see the next section for the detailed description of the substitutions). However, the “wild-type” nucleotides at the sites of replacements, as found in the reference sequences (*Tca*, XP_973636; *Ptr*, Broad Institute PTTG_05472.1; *Tae* TPA_BK008647), were confirmed at least once in the sequenced cDNAs, in support of the authenticity of the reference sequences. Accordingly, the T-vector clones with the least amino acid substitutions were chosen for expression studies as described next.

For expression in yeast, the amplified genes were cloned under a *GAL*-dependent promoter to express FLAG-epitope-tagged proteins. To clone 5MP genes into pEMBLyex4-based high copy (hc) *GAL*-dependent expression vector, the ~1.2-kb MluI-BamHI DNA fragments carrying *Tca* (clone 1)*, Tae* (clone SB5), and *Ptr* (clone 7) 5MP ORFs and the MluI-BglII fragment carrying *Gin* 5MP ORF were subcloned into the MluI-BamHI sites of pAV1427, generating pEMBL-Tca 5MP, pEMBL-Tae 5MP, pEMBL-Ptr 5MP, and pEMBL-Gin 5MP. Yeast transformants carrying these plasmids express FLAG-tagged 5MP in galactose media.

**Sequences of 5MP-coding DNA from *T. castaneum*, *T. aestivum*, and *P. triticina***

As shown in Table S5, *Tca* 5MP and *Tae* 5MP-coding DNAs display relatively high replacement rates, 0.22% and 0.61%, respectively, as measured by the total number of replacements divided by the total number of bases sequenced. This is much higher than the experimental error rate of 0.04%, as calculated from the experiments with *Gin* 5MP whose RNA was isolated from a homogeneous population of the strain WB (of the two T-vector clones sequenced, one had no replacement and reported as KC196541, while the other had a single base substitution). Therefore, most of the replacements found in *Tca* 5MP and *Tae* 5MP cDNAs, and especially those found twice or more in different clones (italicized in Tables S2 and S3), are likely to be authentic polymorphisms. This is in agreement with the fact that *Tca* RNA was isolated from several pupae from unidentified parents. While we used wheat leaves from a single plant to prepare *Tae* cDNAs, *T. aestivum* is a hexaploid (AA BB DD), with six sets of chromosomes, two sets from each of three different species. Therefore, the T16 5MP clone sequence with 9 synonymous replacements and 3 nonsynonymous replacements (Table S3) may be derived from the chromosome distinct from those coding for other 5MP sequences.

In the case of the wheat rust *P. triticina*, RNA was derived from a single race (strain), MHDS. The two replacements, *C714T (A239A)* and *C728A (A243E),* found in all clones, are polymorphisms that had been confirmed from genome sequencing of this race. Three clones (# 4, 7 and 11) have no other replacements, confirming the genome sequencing (Table S4). However, the cDNA sequences from this species displayed a replacement rate (0.11%), slightly higher than the experimental error rate calculated with *Gin* 5MP DNA (Table S5). Of note were the two clones (# 2 and 10) carrying a stop codon mutation, a clone (# 1) carrying a base deletion, and a clone (#6) with an unspliced intron, none of which was observed in the experiments with other species. To see if the *Ptr* cDNA for another gene contains these types of mutations, we cloned eIF5-coding DNA. As shown in Table S5, *Ptr* eIF5 did not display such deleterious changes, with a low rate of replacement (0.06%), equivalent to the experimental error rate (3 of the 7 clones isolated had no replacements compared to the reference sequence). RNA-seq analysis of *P. triticina* indicates that alternative splicing is prevalent in this organism and confirms the presence of 5MP mRNA with the unspliced intron found in clone 6. Therefore, it is possible that the lethal changes observed with 5MP are the results of post-transcriptional modification of a subset of rust genes in infected leaves. Since the RNA-seq is not designed to detect posttranscriptional base changes made *e. g.* by RNA editing, further work such as conventional EST sequencing is required to test this idea.

**Biochemical and genetic studies in yeast *Saccharomyces cerevisiae*.**

Yeast strains KAY33 (*gcn2Δ GCD6^+^ ura3 HIS4-lacZ*) and KAY34 (*gcn2Δ gcd6-7A ura3 HIS4-lacZ*) (9) were transformed with 5MP expression plasmids together with a vector control. Transformants of KAY33 were grown in SCGal-ura medium for 6-7 hrs at 30^o^C to induce *GAL*-dependent transcription (10). The cells were harvested to prepare whole cell extracts (WCE) by glass beads disruption (11). Fixed amounts of WCE were subjected to immunoblot analysis with anti-FLAG and anti-Tubulin (loading control) antibodies. The FLAG-5MP protein complexes with yeast proteins were affinity-purified with M2 anti-FLAG affinity resin from WCE prepared from KAY33 transformants, and eluted with FLAG peptides (Sigma), as described (1). Equal portions of eluates and WCE from the transformant expressing *Tca* 5MP were analyzed by immunoblotting with anti-FLAG, anti-Gcd11, anti-Sui2, anti-Tif35, anti-Tif5 and anti-RpsO antibodies.

The *gcd6-7A* allele in KAY34 decreases eIF2 activity due to defective guanine nucleotide exchange for eIF2. As a consequence, 5MP expression inhibits eIF2 activity enough to increase *GCN4* expression and confer 3AT resistance in the *gcn2Δ* background. 3AT sensitivity of KAY34 transformants was assayed by spotting 5 μl of yeast culture diluted to A_600_=0.15 and its 10-fold serial dilutions onto SCGal-ura-his plates with or without 60 mM 3AT. *HIS4-lacZ* expression that is controlled under Gcn4p was assessed by β-galactosidase assay (10).

**Purification of recombinant human 5MP1 proteins.** Plasmids to express and purify human 5MP1 in bacteria are constructed as follows. The 0.6-kb EcoRI-NotI fragment of pKA1156 (KA, personal collection) encompassing the coding region for 5MP1-CTD (aa. 245-419) was cloned into pET28c (Novagen). The resulting plasmid was digested with EcoRI and NdeI, to remove the N-terminal T7 eptitope-tag coding region, followed by filling-in with Klenow fragment and self-ligation, generating pET-h5MP1-W2. The 0.74-kb or 1.3-kb EcoRI-NcoI fragments of pKA1144 (KA, personal collection) and pGEX-h5MP1 (pKA1477) (1), were cloned into the following vector pKA1476 to generate pET-h5MP1-MA3 encoding h5MP1-NTD (aa. 1-245) and pET-His-h5MP1 encoding the full-length h5MP, respectively. pKA1476 was constructed by digesting pET28c with BamHI and NdeI, followed by filling-in with Klenow enzyme and self-ligation.

*E. coli* BL21 (DE3) transformats carrying these plasmids were grown at 37^o^ C in LB-Kan medium to an OD_600_=0.6, cooled to 23^o^ C, and induced for T7 RNA polymerase expression with 0.5mM IPTG overnight. Cells were harvested and bacteria pellet stored at -20^o^ C. To purify the recombinant h5MP1 proteins, the bacteria pellets were resuspended to 40-ml of a buffer containing 20mM HEPES (pH 7.5), 100mM NaCl, 0.1% Triton-X, lysozyme, benzonase, 2mM TCEP, and lysed by sonication. The lysate was centrifuged at 40000g for 1hr and the supernatant passed through a 0.25μm filter. The cleared lysate was then adjusted to a final concentration of 500mM NaCl, 10% Glycerol and 50 mM imidazole, applied to 5 ml Ni-NTA agarose beads column pre-equilibrated with a similar buffer and left in a rocking platform at 4^o^ C for 45 min. The flow through was discarded and the beads mixed, washed 10 times with 20 ml of a wash buffer containing 500 mM NaCl, 10mM HEPES (pH 7.5), 0.5mM TCEP, 10% Glycerol, and 50mM imidazole. The proteins were eluted in ten 5-ml fractions with an elution buffer with the same content as the wash buffer except that the addition of imidazole is 300 mM. The elution fractions were pooled and concentrated by ultrafiltration through a 10-kDa cutoff filter to a final volume of 4 ml. Concentrated proteins were passed through a Superdex-75 size exclusion column pre-equilibrated to a buffer consisting of 10 mM HEPES (pH 7.5), 125 mM NaCl, 0.5 mM TCEP and appeared as a single peak in the correct volume. Fractions were pooled and further concentrated by ultrafiltration to about 5 mg/ml at which point they were flash frozen in liquid nitrogen and kept at -80^o^ C until used for ITC. Recombinant h5MP1 proteins gave a good yield of 20-40 mg/l culture, compared to ~5 mg/l culture for eIF2β _53-136_.

**Examination of uORFs in ATF4 mRNA leader region in Metazoa.**

ATF4 homologs were found by reiterated PSI-BLAST searches with *D. melanogaster* Cryptocephal isoform a (Crc-A) (12). Its homolog in *Metaseiulus occidentalis* (mite) was obtained by reiterated PSI-BLAST searches with this protein against arthropod data but excluding insect data. Its homolog in mollusk and hydra were obtained by reiterated PSI-BLAST searches with *C. elegans* ATF-5 against metazoa data excluding vertebrates, arthropods and nematodes. While most of the identified ATF4 mRNA sequences contain the coding sequence only, the following sequences included 5’ UTR sequences and were reported here in Fig. 3. Accession numbers for arthropod species are *T. cas*, XM_961423; *B. imp*, XM_003489056; *P. xut*, AK401298; *C. cap*, XM_004521424; *D. mel*, NM_080158; *M. occ*, XM_003746093. Accession numbers for other metazoan species are *C. ele*, CAA93757; *A. cal*, NM_001204701; *L. sta*, AB083656; *B. gla*, FJ807771; *C. ari*, EF405960; *H. mag*, XM_002160108.

**Cloning of *Tca ATF4* cDNA**

We used *Tca* *ATF4* mRNA sequence (XM_961423) to design oligos, Tca ATF4 5’ Hd and Tca ATF4 3’, corresponding to the 5’ end of its 5’ UTR and the 3’ end of the coding region, respectively, as listed in Table S1. We PCR-amplified and cloned ATF4 cDNA using these oligos and *Tca* pupal cDNA as template. Four T-vector clones were sequenced. Nucleotide replacements found in these clones are listed in Table S7 under “longer constructs”. The results confirmed the 195-bp UTR sequence 5` of the *ATF4* start codon, including uORF1 and uORF2 as found in the genome sequence (Fig. 3A). None of the sequence replacements found in this area changes the length of these uORFs (Table S7). The A(-3)-to-G replacement changes the Kozak consensus for *ATF4* start codon, but does not change its strength (A and G are equally preferred at the position -3).

For *Tca ATF4* RNAi construction, we similarly cloned cDNA just corresponding to *ATF4* ORF using oligos Tca ATF4-F and Tca ATF4 3’. We chose the clone 7 that did not have any nucleotide replacement except for a polymorphism found in all the sequenced beetles (Table S7, shorter constructs) and contained T7 promoter sequence 5’ of the insert as template for production of dsRNA targeting the 5’ half of ATF4 ORF. To produce dsRNA targeting its 3’ half, the 0.66-kb EcoRI-XhoI fragment of ATF4 clone 7, encompassing 3’ two thirds of *ATF4* ORF, was cloned into the EcoRI-SalI sites of pGEM-T Easy to generate pTca ATF4-7R (pKA1586). The T7 promoter sequence in the vector is located 3’ of the *ATF4* insert in this clone.

**RNA-interference studies in *Tribolium castaneum***

dsRNA fragments, designed from two different regions of the *T. castaneum* gene of interest, were prepared with T7 RNA polymerase and PCR-derived DNA template, essentially as described (13). We took advantage of the T7 promoter sequence, which flanks the multiple cloning site of pGEM-T vectors. To generate RNAi constructs for knocking down 5MP, we used T-vector *Tca* 5MP clones 1 and 4 (Table S2) as template for PCR with the T7 promoter sequence, located 5’ and 3’, respectively, to the 5MP ORF DNA. Then PCR was conducted, using *Taq* DNA polymerase (Promega GoTaq^TM^ polymerase), with oligos T7-1 and Tca 5MP dsRNA-616 and with oligos T7-1 and Tca 5MP dsRNA-690 (Table S1), respectively, to generate the template for T7 transcription. Using this DNA as template and the MEGAscript T7 Kit (Ambion), we prepared 5MP dsRNA-616 and 5MP dsRNA-690, covering nt 1-616 and 690-1269, respectively, of the *Tca* 5MP ORF. To generate RNAi constructs for ATF4 knockdown, we similarly performed PCR with oligos T7-1 and Tca ATF4 dsRNA-477 and T-vector Tca ATF4 clone 7 (Table S7) as template and with oligos T7-2 and Tca ATF4 dsRNA-478 and pTca ATF4 clone 7R as template, in order to generate Tca ATF4 dsRNA-477 (*ATF4* ORF nt. 2-477) and Tca ATF4 dsRNA-478 (*ATF4* ORF nt. 478-957), respectively. Since we failed to generate the transcription template for Tca ATF4 dsRNA-478 when we used the Taq DNA polymerase, we used a high-fidelity enzyme, Vent DNA polymerase (NEB), to generate this template at a sufficient yield.

The resulting dsRNA was injected at fixed concentrations (50 ng – 2 μg/μl) into groups of adult females of the same number (25~50), as described previously (13). Additionally, the same number of beetles were injected with buffer only. Adult survival was determined after 24 hours, and at each egg collection. Beginning 3 or 4 days after injection, eggs were collected, counted, and allowed to develop in a jar containing an appropriate medium. This was done in alternating 3 and 4 day intervals for three weeks. Insect development was assessed by counting the number of larvae, pupa and adults for every jar once every week until all the insects in the jar became adults. Rates of hatching and survival to pupal and adult stages were documented based on these data.

**Real-Time PCR**

*T. castaneum* adult, egg, or larval RNA was purified with RNeasy columns (Qiagen, catalog no. 74104), as described above for pupal RNA, except that eggs were dechorinated prior to freezing in liquid nitrogen. 1~3 μg RNA was used to prepare cDNA with Superscript^TM^ III First-strand synthesis system (Invitrogen). Quantitative PCR was performed in triplicate to evaluate mRNA levels by using iQ SYBR Green supermix (Bio-rad) and gene-specific primers listed in Table S1 on a CFX96 Real-Time System (Bio-rad). In the case of RNA isolated from 0-12 and 12-24 hr eggs (~20 eggs at a time),, quantitative PCR was performed only in duplicate due to low yields of RNA (0.2~1 μg total) (all RNA isolated were used for a single cDNA synthesis for one set of RT-PCR).

**Reference:**

***Table S1. Oligonucleotides used in this study***

| Name | Sequence (5’ to 3’, with restriction sites underlined) | Description |
| --- | --- | --- |
| Ptr 5MP 5’ | ACG CGT CAT *ATG* TCT GCC GCT GCT GCT GC | *Puccinia triticina* 5MP ORF nt. 1 to 20 (start codon is italicized) |
| Ptr 5MP 3’ | GCG GCC GCG GAT CC*T* *CA*C TCA TCA TCA TCA TCG TCC | *Puccinia triticina* 5MP ORF nt.1269 to 1248 (stop codon is italicized)) |
| Tca 5MP 5’ | ACG CGT CAT *ATG* AGT CAA AAA GTA GAA AAA CCA G | *Tribolium castaneum* 5MP ORF nt. 1 to 25 (start codon italicized) |
| Tca 5MP 3’ | GCG GCC GCG GAT CC*C* *TA*C TCA GTG CTG CCT TCA GAA TC | *Tribolium castaneum* 5MP ORF nt. 1269 to 1246 (stop codon italicized) |
| Tae 5MP 5’ | ACG CGT CAT *ATG* AGC TCG AAG GAG AAG CCC | *Triticum aestivum* 5MP ORF nt. 1 to 21 (start codon italicized) |
| Tae 5MP 3’ | GCG GCC GCG GAT CC*T TA*T TCT TCC TCC TCT GCC TCC | *Triticum aestivum* 5MP ORF nt. 1239 to 1218 (stop codon italicized) |
| Gin 5MP 5’ | GCA T*AT G*AC GCG TTC GAA GCC TCT CCC TGG | *Giardia intestinalis* 5MP ORF nt. 4 to 20 (start codon italicized) |
| Gin 5MP 3’ | GGA TCC AGA T*CT A*GT CCT CTC CAT CGT CG | *Giardia intestinalis* 5MP ORF nt. 1233 to 1215 (stop codon italicized)) |
| Tca 5MP dsRNA-616 | *TAA TAC GAC TCA CTA TAG GG*A ATT TAC ACC CTT GTC C | *Tribolium castaneum* 5MP ORF nt. 616 to 597 (T7 promoter italicized) |
| Tca 5MP dsRNA-690 | *TAA TAC GAC TCA CTA TAG GG*T GTA CTT CCG TAA CGC C | *Tribolium castaneum* 5MP ORF nt. 690 to 708 (T7 pomoter italicized) |
| Tca ATF4 dsRNA-477 | *TAA TAC GAC TCA CTA TAG* *G*GG AGC TGG AGG AGC G | *Tribolium castaneum* ATF4 ORF nt. 477 to 461 (T7 promoter italicized) |
| Tca ATF4 dsRNA-478 | *TAA TAC GAC TCA CTA TAG G*TT GTA CCT GAA AAG CTG GC | *Tribolium castaneum* ATF4 ORF nt. 478 to 497 (T7 promoter italicized) |
| Tca ATF4 5’ Hd | aag ctt TTT CTC CTC GGC TCA TCG | *Tribolium castaneum* ATF4 5’ UTR nt. – 192 to – 175 |
| Tca ATF4 3’ | gga tcc *TTA* GTT GAT AAG TCC CTT TGC | *Tribolium castaneum* ATF4 ORF nt. 957 to 937 (stop codon italicized) |
| T7-1 | TAATACGACTCACTATAGGG | Matching to T7 promoter |
| T7-2 | TAATACGACTCACTATAGG | Matching to T7 promoter |
| Tca 5MP-F | CCA GTT CTA TCC GGT CAA CG | *Tribolium castaneum* 5MP ORF nt. 22 to 41 |
| Tca 5MP-R | GGT CCT TGA CCA AGT GCG AA | *Tribolium castaneum* 5MP ORF nt. 550 to 531 |
| Tca ATF4-F | TGA GTT GTG CGC CGT TCA TT | *Tribolium castaneum* ATF4^a^ ORF nt. 2 to 21 |
| Tca ATF4-R | GGT ACG CCA GCT TTT CAG GT | *Tribolium castaneum* ATF4^a^ ORF nt. 502 to 483 |
| Tca GADD34 F | CAA CAG AAG AGT GCA AGA CAG C | *Tribolium castaneum* GADD34^b^ ORF nt. 277 to 298 |
| Tca GADD34 R | GCC TCA GTA TTG CGT ACA CGT T | *Tribolium castaneum* GADD34^b^ ORF nt. 566 to 545 |
| Tca RPS3-F | ACC TCG ATA CAC CAT AGC AAG C | *Tribolium castaneum* RPS3^c^ ORF nt. 365 to 352 (14) |
| Tca RPS3-R | ACC GTC GTA TTC GTG AAT TGA C | *Tribolium castaneum* RPS3^c^ ORF nt. 188 to 209 (14) |

^a^ XM_961423 ; ^b^XM_962338; ^c^ [XM_965494](http://www.sciencedirect.com/science?_ob=RedirectURL&_method=externObjLink&_locator=ncbi-n&_issn=01677012&_origin=article&_zone=art_page&_plusSign=%2B&_targetURL=http%253A%252F%252Fwww.ncbi.nlm.nih.gov%252Fentrez%252Fquery.fcgi%253Fcmd%253Dsearch%2526db%253Dnucleotide%2526doptcmdl%253Dgenbank%2526term%253DXM_965494)

**Table S2. Nucleotide replacements found in 5MP clones isolated from *T. castaneum***

| Clone# | Replacement # | Acc# |
| --- | --- | --- |
| 1 | T528C (S176S), *A618G (L206L),* **A919G (I307V),** *G927A (E309E),* **T1190C (F397S)** | KC261419 |
| 2 | *A618G (L206L),* **A814G (N272D)**, *G927A (E309E),* **T992C (L331S)** | KC261420 |
| 4 | **A56G (K19R), A1204G (E402G)** | KC261421 |
| 5 | **G1178C (G393A)** | KC261422 |
| 6 | **T806C (L269P), A1061G (Y354C)** | KC261423 |

# Nucleotide replacements in bold font change amino acids. Others are synonymous replacements. Replacements found twice or more are italicized.

**Table S3. Nucleotide replacements found in 5MP clones isolated from *T. aestivum***

| Clone# | Replacement # | Acc# |
| --- | --- | --- |
| SB5 | **T196C (F66L),** *C343T (L115L),* *A435G (Q145Q),* **A638G (D213G),** *A1047G (Q349Q),* *T1215C (L405L*) | JX978404 |
| T5 | **T163C (S55P),** **T209C (V70A),** *C343T (L115L),* *A435G (Q145Q)*, **T530C (V177A)**, T780C (T260T), *A1047G (Q349Q),* **A1103G (Y368C)**, *T1215C (L405L)* | JX978405 |
| T8 | **T74C (L25S), T110C (I37T), A1187G (K396R)** | JX978406 |
| T16 | **G130A/C132T (D44N)**, *C343T (L115L),* G369A (K123K), A393G (K131K), T405C (S135S), *A435G (Q145Q),* G546T (V182V), **G737C (S246T),** *A1047G (Q349Q),* A1098C (S366S), G1197A (E399E) | JX978407 |

# Nucleotide replacements in bold font change amino acids. Others are synonymous replacements. Replacements found twice or more are italicized.

**Table S4. Nucleotide replacements found in 5MP clones isolated from *P. triticina***

| Clone# | Replacement # | Acc# |
| --- | --- | --- |
| 4, 7,11 | *C714T (A239A)*,* ***C728A (A243E)**** | KF176343 |
| 1 | **ΔT594**, *C714T (A239A)*,* ***C728A (A243E)****, T990C (A330A), **T1015C (C339R**) |  |
| 2 | **A445T (K149 STOP)**, *C714T (A239A)*,* ***C728A (A243E)*,* G1186A (A396T)** |  |
| 3 | **T578C (F193S), A688G (K230E)**, *C714T (A239A)*,* ***C728A (A243E)*****,* A864G (Q288Q), **G1066A (A356T)** | KF176342 |
| 6 | **Third intron unspliced** between G380 and A381, *C714T (A239A)*,* ***C728A (A243E)**** |  |
| 8 | T705C (D235D), *C714T (A239A)*,* ***C728A (A243E)**** | KF176344 |
| 9 | **A437G (Y146C)**, *C714T (A239A)*,* ***C728A (A243E)**** | KF176345 |
| 10 | A135G (L45L), *C714T (A239A)*,* ***C728A (A243E)*****,* **C1192T (Q398 STOP)** |  |

# Nucleotide replacements in bold font change amino acids. Others are synonymous replacements. Replacements found twice or more are italicized.

* Polymorphism found in race MHDS.

Table S5. Replacement rates of 5MP-coding regions observed in this study

| Protein | *Gin* 5MP | *Tca* 5MP | *Tae* 5MP | *Ptr* 5MP | *Ptr* eIF5# |
| --- | --- | --- | --- | --- | --- |
| ORF length (bp) | 1233 | 1269 | 1239 | 1269 | 1209 |
| # of clones sequenced | 2 | 5 | 4 | 10 | 7 |
| # of replacements found | 1 | 14 | 30 | 14* | 5 |
| Replacement rate (%) | 0.040 | 0.220 | 0.605 | 0.110 | 0.059 |

* Excludes race-specific polymorphisms, and includes a base deletion and the mis-splicing of an intron (see Table S4).

# Genbank acc. # KF176341.

**Table S6. Possible eIF3 subunits found in *G. intestinalis***

| subunit | Strain WB (ATCC50803) | Strain P15^e^ | ATCC 50581^e^ | % ID to *S. cerevisiae* homolog^f^ |
| --- | --- | --- | --- | --- |
| eIF3b^a^ | XP_001708681 | EFO64742  (91%) | EES98635 (78%) | 20 % (aa 40-291) |
| eIF3c^b^ | XP_001704399 | EFO65587 (90%) | EET00954 (77%) | 24 % (aa 675-759) |
| eIF3g^c^ | XP_001708614 | EFO61407 (83 %) | EES99585 (63 %) | 21 % (aa 53-173) |
| eIF3i^d^ | XP_001705299 | EFO62874 (88%) | EES98952 (73 %) | 24 % |

^a^Identified by reiterated PSI-BLAST search with *S. cerevisiae* eIF3b (Prt1p). Reiterated PSI-BLAST search with the WB strain protein identifies many other fungal eIF3b at 24-20 % identity.

^b^Identified by reiterated PSI-BLAST search with *S. cerevisiae* eIF3c (Nip1p). Reiterated PSI-BLAST search with the WB strain protein identifies eIF3c from *Neospora caninum* (XP_003879699) between aa 545 and 787 at 22 % identity.

^c^Contains “Eukaryotic translation initiation factor 3 subunit G” motif (pfam12353) at aa. 16-146. Reiterated PSI-BLAST search with the WB strain protein identifies a protozoan (*Monosiga brevicollis*) and fungal eIF3g at 22-20 % identity.

^d^Annotated as “eukaryotic translation initiation factor 3 subunit 2”

^e^% ID to the WB strain protein is presented in parentheses.

^f^Region of *S. cerevisiae* protein amino acids identified by the reiterated BLAST search with the WB strain protein is listed in parentheses.

**Table S7. Nucleotide replacements found in ATF4 clones isolated from *T. castaneum***

| Clone# | Replacement # | Acc# |
| --- | --- | --- |
|  | Longer constructs (with 5’ UTR) |  |
| 4 | *T-56C (A17A*)*, **A149G(E50G)**, *G705A (R235R)*, A936G (K312K) | KJ405474 |
| 13 | T-127C, *T-56C (A17A*),* ***A-3G (Q35R*)****,* **A401G (E134G)**, **T494A (L165Q)**, *G705A (R235R)* | KJ405475 |
| 14 | *T-56C (A17A*),* ***A-3G (Q35R*)****,* *G705A (R235R)*, **T929C (L310P)** | KJ405476 |
| 17 | *T-56C (A17A*),* ***A-3G(Q35R*)****,* *G705A (R235R)* | KJ405477 |
|  | Shorter constructs |  |
| 7 | *G705A (R235R)* | KJ405472 |
| 8 | *G705A (R235R)*, **T917C (L306P)** | KJ405473 |

# Nucleotide replacements in bold font change amino acids. Asterisk (*) shows amino acid change in uORF2. Others are synonymous replacements. Replacements found twice or more are italicized.

**Legends (Supplementary figures)**

**Fig. S1. Sliding window analyses of pairwise 5MP1 and 5MP2 d*N*/d*S*.** (A) The pairwise comparisons were made between *Gallus gallus* (gg) and *Homo sapiens* (hs) 5MP1 and 5MP2, respectively, and were performed using a window of 100 and jump of 10. (B) The pairwise comparisons were made between 5MP1 and 5MP2 in the same species: *Drosophila virilis* (dvir) and *Drosophila mojavensis* (dmoj) and were performed using a window of 100 and jump of 10.

**Fig. S2. Effect of 5MP RNAi on time for larvae to become pupae (repeat injection).** 5MP dsRNA-616 and -690, designed from two different regions of the 5MP gene, were injected at 1μg/μl into 28 adult females each. 125 eggs were collected 4, 8 and 11 days after injection in total and placed on culture flour to assess larval development. The line graph indicates the number of larvae which were produced from mock-injected adults (as control) and became adults in the indicated times. The gray arrows indicate data for larvae produced from adults injected with indicated 5MP RNAi constructs. Because of the sample size, only a small number of larvae became adults when they are derived from adults injected by 5MP RNAi. However, their pupation time is longer than average (data shown in black) for control experiments. The remainder of the larvae were fixed in paraformaldehyde for staining with anti-horseradish peroxidase or anti-5MP mRNA to assess embryonic development (see text).

**Fig. S3. Effect of ATF4 RNAi on larval development.** ATF4 dsRNA-477 and -478, designed from two different regions of the ATF4 gene, were injected at 50 ng/μl and 1μg/μl into 40 adult females each. 100 eggs were collected 3, 6, 10 and 14 days after injection and placed on culture flour to assess larval development. The hatch rate for the total of 400 eggs was somewhat low (~30-40%) regardless of the RNAi treatment, although, initially, ATF4 dsRNA-477 treatment reduced the number of hatches (see panel A below). We are not sure if this is due to RNAi treatment, since this was not observed with dsRNA-478 and since the hatch rate was generally low in this experiment. (A) and (B) The graphs indicate the average time (in day since egg lay) it takes for larvae that hatched from eggs collected 3 days (A) or 3 and 6 days after RNA injection combined (B) to pupate. Bars indicate SEM. The numbers across the top indicate the number (n) of larvae that hatched in each egg collection. The numbers at the bottom summarize the p value for the change in the average pupation time (computed from >40 larvae) caused by each RNAi treatment (shown by arrows), compared to larvae from buffer-treated adults. (C) A summary of the number of larvae showing unusually long pupation time (column 2) or unusually long survival time prior to premature death (column 3). Column 1 indicates the population showing normal pupation time. The numbers in parentheses indicate the percentage compared to the total number of larvae that became pupae.
